# Evaluation and Validation of Thorax Model Responses: A Hierarchical Approach to Achieve High Biofidelity for Thoracic Musculoskeletal System

**DOI:** 10.3389/fbioe.2021.712656

**Published:** 2021-07-16

**Authors:** Wei Zeng, Sayak Mukherjee, Adrian Caudillo, Jason Forman, Matthew B. Panzer

**Affiliations:** Center for Applied Biomechanics, University of Virginia, Charlottesville, VA, United States

**Keywords:** injury biomechanics, musculoskeletal system, finite element method, thorax model, validation, biofidelity, thoracic spine

## Abstract

As one of the most frequently occurring injuries, thoracic trauma is a significant public health burden occurring in road traffic crashes, sports accidents, and military events. The biomechanics of the human thorax under impact loading can be investigated by computational finite element (FE) models, which are capable of predicting complex thoracic responses and injury outcomes quantitatively. One of the key challenges for developing a biofidelic FE model involves model evaluation and validation. In this work, the biofidelity of a mid-sized male thorax model has been evaluated and enhanced by a multi-level, hierarchical strategy of validation, focusing on injury characteristics, and model improvement of the thoracic musculoskeletal system. At the component level, the biomechanical responses of several major thoracic load-bearing structures were validated against different relevant experimental cases in the literature, including the thoracic intervertebral joints, costovertebral joints, clavicle, sternum, and costal cartilages. As an example, the thoracic spine was improved by accurate representation of the components, material properties, and ligament failure features at tissue level then validated based on the quasi-static response at the segment level, flexion bending response at the functional spinal unit level, and extension angle of the whole thoracic spine. At ribcage and full thorax levels, the thorax model with validated bony components was evaluated by a series of experimental testing cases. The validation responses were rated above 0.76, as assessed by the CORA evaluation system, indicating the model exhibited overall good biofidelity. At both component and full thorax levels, the model showed good computational stability, and reasonable agreement with the experimental data both qualitatively and quantitatively. It is expected that our validated thorax model can predict thorax behavior with high biofidelity to assess injury risk and investigate injury mechanisms of the thoracic musculoskeletal system in various impact scenarios. The relevant validation cases established in this study shall be directly used for future evaluation of other thorax models, and the validation approach and process presented here may provide an insightful framework toward multi-level validating of human body models.

## Introduction

The human thorax is a fairly complex body region with musculoskeletal structures (ribcage) and soft tissue organs in the thoracic cavity. The ribcage consists of 12 thoracic vertebrae (T1–T12), 12 pairs of ribs, costal cartilages, sternum, scapulae, and clavicles. In automobile crashes, thoracic injury is one of the most prevalent injuries, and ranks second only to head injury with reference to the number of fatalities and serious injury outcomes (Forman et al., 2019). According to the Abbreviated Injury Scale (AIS), an anatomically based injury severity scoring system, the musculoskeletal (MSK) injury accounted for the highest percentage of thoracic AIS in all passenger vehicle occupants (Cavanaugh and Yoganandan, 2015). The thoracic MSK system protects intrathoracic structures from external penetration or blunt trauma, which is the leading cause of injury during motor vehicle collisions (MVC). Biomechanically, the external force under blunt impact is mostly sustained by the bony skeleton of the thorax, and the impact energy can be dissipated by muscles and soft organs with viscoelasticity properties. A better understanding of the thorax biomechanics, especially the biomechanical response and injury mechanisms of thoracic MSK, is crucial to prevention and mitigation of thorax injuries.

To investigate MVC related human injuries and enhance vehicle safety designs, surrogates of occupants have been used, including post mortem human surrogates (PMHSs), anthropomorphic test devices (ATDs), and volunteers. They each have their strengths and limitations, but their common feature is that they provide a similar response to that of the living human body during a crash and are studied to assess MVC injuries and develop or improve design for automotive safety systems (Beeman et al., 2012). Over the past three decades, computational finite element (FE) human body models (HBMs) have emerged as a powerful and versatile tool that can accurately describe the human anatomy, biomechanics, and variability for injury risk predictions and vehicle safety systems development. Compared to surrogates for safety designs, computational thorax FE models can integrate and represent thoracic biomechanical data from PMHS or volunteers, as well as efficiently evaluate both overall thoracic responses and localized physical variables related to injuries. For example, FE models can quantitatively predict complex chest deformation and bone fracture patterns that are not able to be evaluated via ATDs (Pipkorn and Kent, 2011).

A considerable number of thorax FE models have been developed to simulate chest behaviors under complex kinematics during various impact loading conditions. Yang et al. (2006) reviewed thorax FE models published before 2005 and after 2005 (Yang, 2018). These models were either isolated thorax models (Deng et al., 1999; Shen et al., 2008) or integrated into full body model (FBM) (Robin, 2001; Haug et al., 2004; Kimpara et al., 2005; Song et al., 2009; El-Jawahri et al., 2010; Pipkorn and Kent, 2011; Antona-Makoshi et al., 2015; Iwamoto et al., 2015; Poulard et al., 2015; Schoell et al., 2015; Zhu et al., 2016). Despite the significant difference of modeling details, the primary goal of these models was to evaluate human thoracic response, understand thoracic injury tolerances, and assess injury risks under various impact loads. Ensuring the biofidelity of these FE models, particularly for the thoracic MSK system, is indispensable for the credibility and utility of the tool for predicting injury to the ribcage and the intrathoracic tissues and organs. In general, one of the major limitations of most of these thorax models was the lack of reasonable validations, mainly due to a significant paucity of available experimental data at multi-levels. For example, if a thorax model was validated under a specific impact condition, its kinematics and injuries might not be valid under different impact loads. Even if the model was globally validated, it is uncertain if it has the capability of predicting thoracic responses and injury risk at a localized level. Additionally, the tissue material parameters are not always available, and most of the experimental datasets at the component or organ level were not prepared for the purposes of numerical simulations and model validation. As such, HBMs have been developed by the Global Human Body Models Consortium (GHBMC), which is a multi-institution collaboration involving industry, government, and academic researchers aiming to develop the most biofidelic computational HBMs for crash safety advancement. Although the GHBMC models contain a significant amount of anatomical details and can provide a basis for further model development and applications, the thoracic region in the detailed GHBMC mid-sized male occupant FE model (M50-O) v4.5 model (released in 2018, the base model used for this study) has inadequacies and limitations, particularly in some important structures of the MSK system. These limitations could be attributed to model simplifications or a lack of material verification, which requires further enhancement to increase accuracy and model biofidelity.

The goal of this study was to report on the hierarchical improvement, evaluation, and validation of the GHBMC M50 thorax model, focusing on the biofidelity of the thoracic MSK system at multi-levels. Specifically, this study focuses on improvements and validations to the thoracic spine, clavicle, sternum, costal cartilage, etc. To our knowledge, FE models for thoracic spine (T-spine) are fewer compared to cervical and lumbar spines. Detailed T-spine models with proper verifications and validations (V&V) using experimental studies are relatively rare, especially for impact scenarios. Aira et al. (2019) developed and validated a simplistic T-spine model using quasi-static loading for adjacent vertebrae and rear hub impact for chest force-deflection response. However, it did not include the state-of-the-art modeling techniques and injury-predictive capabilities that have been used in C-spines models (Panzer et al., 2011). This study builds on these previous efforts to enhance the existing GHBMC T-spine model with improved biofidelity, additional validations, and injury simulation capability. The enhanced model was validated against several experimental cases at segment, functional spinal unit (FSU) and full torso levels. The T-spine was incorporated into the thorax model, and it was able to predict the soft tissue deformation and ligament injury response under traumatic loading levels. The costovertebral joints were evaluated at a component level across the whole ribcage. These substructures were validated for multiple directions of motion, including ventral-dorsal and cranial-caudal flexion. For bony skeleton tissues, the material parametric studies were implemented to determine the properties of the clavicles, sternum, and costal cartilages. Different failure thresholds using maximum principle strain (MPS) were added to these structures to characterize the bone failure features. Several experimental setups were accurately replicated to simulate the axial compression of clavicle, bending testing of clavicle and sternum, and cantilever-beam like bending testing of costal cartilages. The bone behavior of the ribs was not included here since they were already well investigated in previous studies (Li et al., 2010; Poulard et al., 2015; Zaseck et al., 2018).

To evaluate the biofidelity of the whole thorax model, body regional level and full thorax or torso level validation tests were conducted across different loading conditions. These tests contained point loading of the eviscerated ribcage, frontal pendulum impacts, shoulder pendulum impact and table-top belt loading tests. The biomechanical response of the thorax was overall deemed biofidelic and the model was found to be in good agreement with the experimental data using both qualitative and quantitative assessments. The validated thorax model, as demonstrated in this study, was able to serve as a valuable tool for safety researchers and automobile designers to predict, prevent, and mitigate thoracic injuries of vehicle occupants. Additionally, this study describes a multi-level, hierarchical evaluation and validation strategy to enhance the biofidelity of other human body models for injury risk prediction and prevention.

## Materials and Methods

### Body FE Model Overview

The GHBMC 50th percentile male (M50) detailed seated occupant (v4.5) FBM was the basis for the improvement, development, and validation in this study. The model was developed for use with the LS-DYNA solver, a commercial non-linear explicit finite element analysis (FEA) program (LSTC, Livermore, CA, United States). The anatomical geometry of the body was reconstructed based on the anthropometric data scanned from a 50th percentile male volunteer (Gayzik et al., 2009). The high quality quadrilateral and hexahedral meshes of the thorax components were generated by an interactive multi-block meshing approach (Li et al., 2010; Poulard et al., 2015). The FBM contained 1,036 parts, which were discretized by 2.19 million of elements and 1.26 million nodes.

In the base thorax model, the bones of thoracic cage were modeled as elasto-plastic materials with strain-rate dependency (^∗^MAT_Piecewise_Linear_Plasticity) in LS-DYNA, including cortical and trabecular bones of the ribs, sternum, and clavicles. The thoracic vertebrae were represented by rigid bodies, and the costal cartilages were modeled as an elastic material. For each thoracic intervertebral disc, the annulus and nucleus pulposus was modeled as a highly compressible Hill foam (Hill, 1979) and an inviscid fluid, respectively. The thoracic spine ligaments were defined as non-linear elastic spring beams without failure properties. A supplemental table was provided to collect the material models and properties for the important parts relevant to the evaluation and improvement in current study (Supplementary Table 1).

The process of the hierarchical improvement, evaluation, and validation of the thorax model is presented in this section (section “Thoracic Intervertebral Joints Evaluation and Improvement,” section “Costovertebral Joints Evaluation and Enhancement,” section “Clavicle Model Improvement and Validation,” section “Sternum Model Improvement and Validation,” section “Costal Cartilage Model Enhancement and Validation,” section “Ribcage or Full Thorax Level Validation Cases,” and section “Model Performance Evaluation”), and a graphical overview of the steps of the study can be found in the Supplementary Figure 1).

### Thoracic Intervertebral Joints Evaluation and Improvement

**FIGURE 1:**
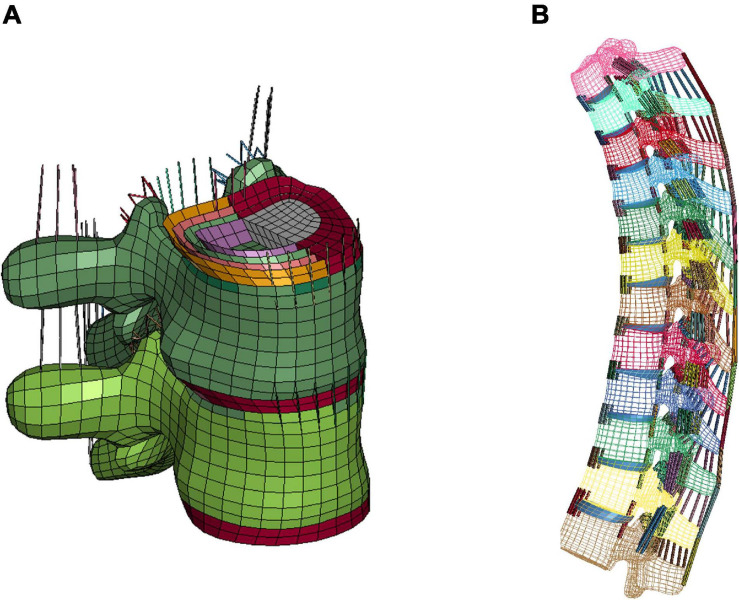
FE modeling of intervertebral discs and ligaments: **(A)** IVD details (T7-T8) and intervertebral ligaments, and **(B)** lateral view of overall thoracic spine model.

The T-spine was enhanced and improved by accurate representation of the components, material properties, and ligament failure properties at tissue level. For the intervertebral discs (IVDs), the nucleus pulposus and annulus fibrosus ground substance were modeled using solid elements. For each IVD, four pairs of concentric quadrilateral layers (8 layers total) were created to represent the annulus fibrosus fiber laminae (Newell et al., 2017), which were embedded in the ground substance (Figure 1A; Panzer and Cronin, 2009). There were 9 major spinous ligaments of the T-spine included in the model, including anterior and posterior longitudinal ligaments, ligamentum flavum, interspinous ligaments, supraspinous ligaments, intertransverse ligaments (left and right), and facet capsular ligaments (left and right) (Figure 1B). The ligament response was characterized using non-linear load-deflection curves identified by three distinct spinal regions (Chazal et al., 1985; Panzer et al., 2011). The failure deflection for each individual ligament was adopted from data reported in Pintar (1986).

**FIGURE 2:**
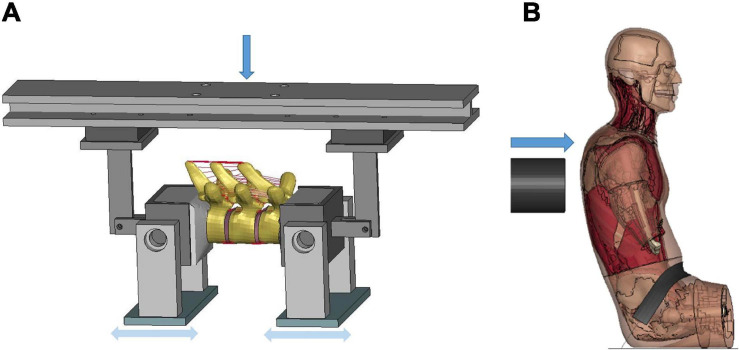
Model setup for flexion bending of FSU and rear hub impact test: **(A)** flexion bending test of FSU T7-T9, and **(B)** hub-impact tests performed on the back surface of the mid-thorax.

Initial quasi-static evaluations of the model were conducted for adjacent vertebrae and the intervertebral disc between them (motion segment) to ensure a valid tissue-level response (Markolf, 1972), including axial tension and compression, shearing and lateral bending. Since the experimental testing of motions segments was not able to represent natural spinal motions due to too many constraints imposed, the FSU with three adjacent vertebrae and two intervertebral discs was therefore adopted by researchers to evaluate more realistic spinal responses (Yang, 2018). In our study, two sets of FSUs (T2–T4 and T7–T9) were simulated (Figure 2A) and compared with experimental testing results of flexion bending (Lopez-Valdes et al., 2011). In addition to the FSU level evaluation, the full torso response and change of spine angle of extension were validated by tests of rear hub impact loading (Forman et al., 2015) with a 97.5 kg impactor using three different velocities: 1.5, 3.0, and 5.5 m/s, shown in Figure 2B.

### Costovertebral Joints Evaluation and Enhancement

The costovertebral joints were evaluated at component level using the method outlined in Duprey et al. (2010). To replicate the experiment setup, the ribs 2, 4, 6, 8, and 10, along with their adjacent vertebrae were isolated from the thorax base model. All relevant ligaments that interconnect the bony structures were preserved for each level of the costovertebral joint, including the radiate, costotransverse ligament, superior costotransverse ligament, lateral costotransverse ligament, and the intra-articular ligament. The radiate was modeled using shells and assigned as a non-linear fabric material. Other ligaments were modeled as tension-only beams with a single constant to define the stiffness. Each rib was inserted by a simple model of a cylindrical rod, which was rigidly constrained to the rib. Four quasi-static rotational motions were applied to the rod to investigate ventral-dorsal and cranial-caudal flexion, as demonstrated in Supplementary Figure 2.

### Clavicle Model Improvement and Validation

**FIGURE 3:**
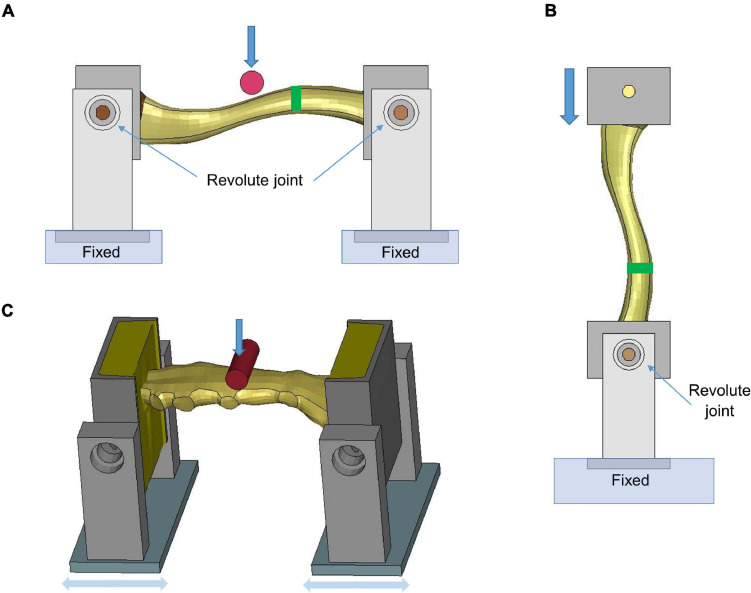
Model setup for clavicle fracture and sternum bending tests: **(A)** three-point bending test (the green rectangle showed the position where the strain gages were attached), **(B)** axial compression test, and **(C)** sternum bending test.

The clavicle model was evaluated at the component level using experimental testing results in Zhang et al. (2014), which loaded the clavicles to fracture by two methods: three-point bending and axial compression, respectively. The FE models were created based on an isolated clavicle from the thorax model and modeling for the testing device. For the three-point bending test, each end of the clavicle was supported by a pinned assembly which allowed rotation along the superior–inferior axis. The impactor in Figure 3A, which was modeled as cylinder with aluminum material properties, was loaded along the anteroposterior direction (i.e., perpendicular to the clavicle longitudinal axis) using the same rate as in the experiments (0.1 m/s). In the axial compression test, as shown in Figure 3B, the medial end was potted into a square-shaped block that allowed rotation, while the lateral end was clamped and moved along the lateral–medial direction by 0.1 m/s. In the experiments, four uniaxial strain gages were attached at the perimeter of the clavicle cross section, as indicated by green rectangles in Figures 3A,B. Based on the force-deflection responses, parametric studies of the material properties were conducted to determine the appropriate elastic modulus and yield strength of the cortical bone. Bone failure was defined using a maximum principal strain (MPS) criterion. Through a brute-force optimization process, it was found that 8% MPS for cancellous bone and 3% MPS for cortical bone produced reasonable results to the experimental data (Duprey et al., 2008). Once the MPS reached the defined threshold, the element was eroded and removed from the calculation.

### Sternum Model Improvement and Validation

The sternum fracture was evaluated under a dynamic three-point bending test (Kerrigan et al., 2010). The test rig was modeled similar to the three-point bending test of the clavicle (Figure 3C). The impactor was put in the middle of the two posts and displaced along the downward direction (1.115 m/s) to represent anteroposterior loading on the sternum. Parametric studies of material properties were conducted to determine appropriate sternum material parameters (elastic modulus, yield strength and failure strain) based on experimental force-time responses.

### Costal Cartilage Model Enhancement and Validation

**FIGURE 4:**
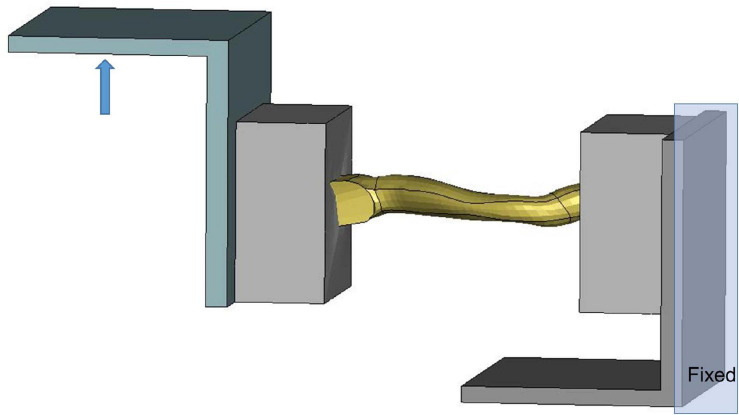
Model setup for costal cartilage bending test.

The costal cartilage model was evaluated at the component level using experimental testing data from Forman et al. (2010). Figure 4 showed the testing device with potted costal cartilage model, which was isolated from the segment connected to the anterior border of the forth rib. The end of cartilage connected to the rib (i.e., right side of the device) was constrained, and the left end was loaded along the vertical direction (0.4 m/s) to represent the situation of mid-chest compression. Anatomically, the costal cartilage is composited of an inner solid of hyaline cartilage and a surrounding tissue layer called the perichondrium. During FE modeling, the solid structure of costal cartilage was updated from a linear elastic material model in base model to an elasto-plastic material with strain-rate dependency. Additionally, a non-linear fabric shell layer that surrounded the solid mesh was incorporated to include the biomechanical contribution of the perichondrium, since it was not explicitly modeled previously. The material parameters were calibrated to the reaction force along the anteroposterior direction between the simulation and the reported experimental data.

### Ribcage and Full Thorax Level Validation Cases

After validating the MSK structures at the component level, the components were integrated into the thorax model in FBM. The overall mechanical response of the chest, which included the contribution of these MSK soft and hard tissues, could be evaluated against a number of loading cases at ribcage or full thorax level. Here four experimental cases were selected based on their relevance, the availability of data, and the large range of structural levels they represented.

#### Point Loading of the Eviscerated Ribcage

**FIGURE 5:**
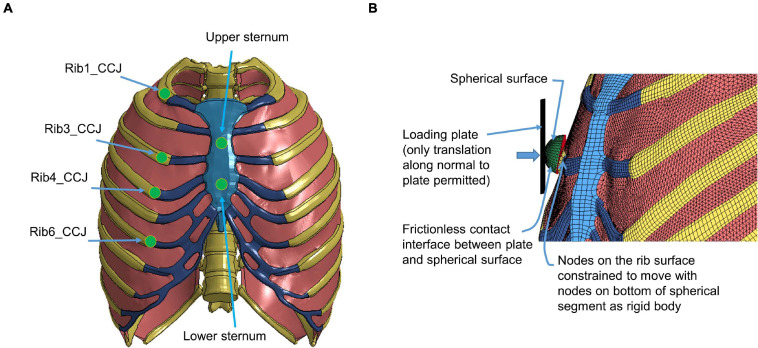
Model setup for point loading simulation: **(A)** illustration of the loading points at the upper and lower sternum levels and at the costochondral junction (CCJ) of rib levels 1, 3, 4, 6, and 9, and **(B)** a close-up at a loading site in the FE model (Kindig et al., 2015).

The quasi-static point loading of the ribcage was simulated using the experimental testing approach reported in Kindig et al. (2010). In the experiment, isolated eviscerated ribcages were mounted upright and quasi-statically loaded by a plate interfacing with a spherical segment glued to the superficial surface of the ribs or the sternum. The reaction force on this plate against applied displacement was measured to check the biomechanical response. To simulate the testing, the FE model of the ribcage (Figure 5) was positioned consistent with the initial position adopted in the experiments. The loading and boundary conditions were also set similarly to the experimental settings: the thoracic vertebrae were constrained and a constant velocity (0.2 m/s) was imposed on the plate up to the prescribed displacement used in the experiment (varied with the point loading site).

#### Frontal Pendulum Impact

**FIGURE 6:**
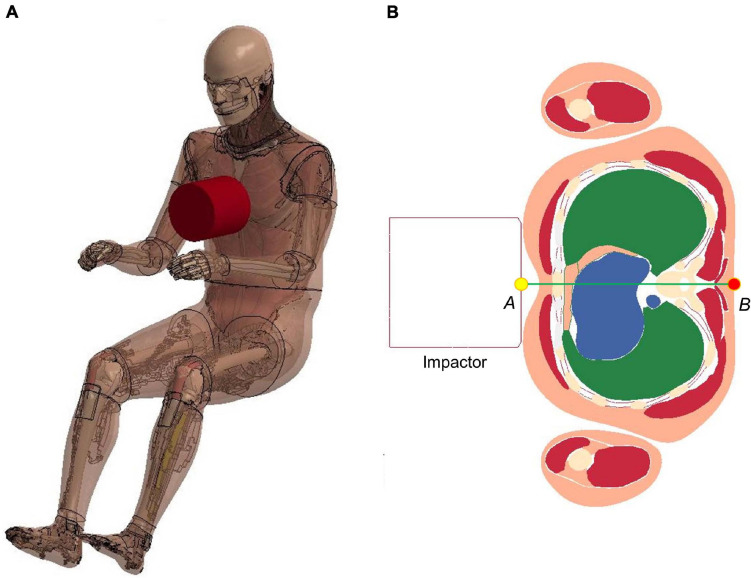
Model setup for frontal pendulum impact simulation: **(A)** illustration of the impact condition. The impact force was measured as the contact force between the impactor and the chest, and **(B)** chest deflection was defined as the change of distance between the center of impactor surface and a node taken on the skin at the T8 level.

The frontal pendulum impact testing was designed to quantify the thoracic response and injury tolerance along the anteroposterior direction under midsternal blunt impact. The PMHS used in the experiments was placed in a seated upright position, and compressed on the frontal chest by a 23.4 kg cylindrical impactor with an initial impact velocity of 4.3 m/s and a diameter of 152 mm centered with the sternum at the level of the 4th intercostal space. In the simulation, the FBM was seated on a rigid plate and the impactor positioned at the same mid-sternum level as in the PMHS testing (Figure 6). The overall thoracic responses (deflection-time and force-deflection relationships) were reported and compared with experimental corridors (Kroell et al., 1971; Lebarbe and Petit, 2012).

#### Shoulder Pendulum Impact

**FIGURE 7:**
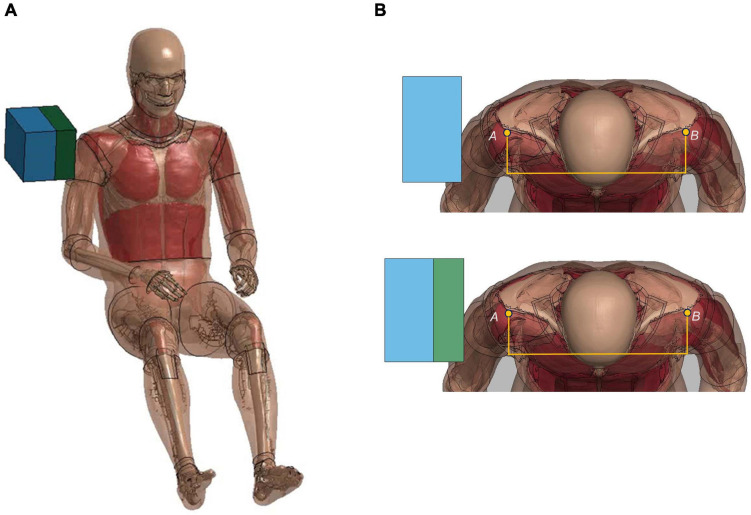
Model setup for shoulder pendulum impact simulation: **(A)** illustration of the impact condition (padded impact), and **(B)** illustration of shoulder deflection measured between points A and B for unpadded and padded simulation cases.

The lateral impact on the shoulder provided response characteristics and injury tolerance of the shoulder and upper thorax along the lateral direction. Koh et al. (2005) presented a meta-analysis of biomechanical responses from several cadaveric studies of lateral impacts on the shoulder, which were developed into biomechanical response corridors for four loading speed cases: 4.4 m/s (foam padded impactor). 4.5 m/s (unpadded), 6.4 m/s (padded), and 6.8 m/s (unpadded). To replicate the experiments, the model was seated on a rigid plate with the arms down by its side (Figure 7A). The impact force was measured as the contact force between the impactor and the thorax. The shoulder deflection was defined as the change of distance between two nodes corresponding to the locations on humeri measured in the experiments (Figure 7B).

#### Table-Top Restraint-Like Loading Tests

**FIGURE 8:**
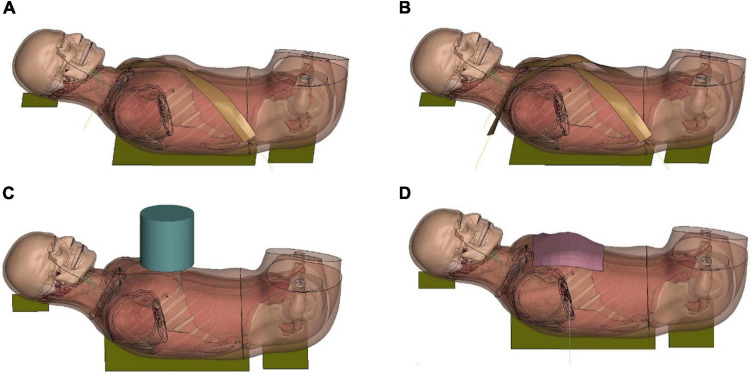
Model setup for table-top loading cases: **(A,B)** showed the single belt and double belts loading (belts were modeled as a shell layer with 2-mm thickness), **(C)** hub loading by a cylindrical rigid hub with a diameter of 152 mm, and **(D)** distributed loading by an extra-wide belt (203-mm width) simulated by a layer of shell elements with 2-mm thickness.

Based on the investigation of the force-deflection response of the thorax under dynamic, non-impact, restraint-like loading at a non-injurious level, Kent et al. (2004) presented testing corridors subjected to single and double diagonal belts and hub and distributed loading on the anterior thorax. The limb amputated PMHS subjects were positioned supine on a table, and then compressed up to 20% of external chest depth for all four loading conditions. Four table-top FE models were set up to simulate the experimental loading cases (Figure 8). The pulleys were modeled using slip ring elements and 1-D belt elements to maintain the loading angles similar to experimental settings. The contact force between the back side of FBM and the table was output as the reaction force. The relative compression was characterized in terms of the ratio of the deflection of a point at the mid-sternum divided by initial distance between this point and the table along anteroposterior direction.

### Model Performance Evaluation

To quantitatively evaluate the model performance, the outputs between model simulations and the experimental testing were compared by a widely used objective rating tool known as CORelation and Analysis or CORA (Gehre et al., 2009; Vavalle et al., 2013; Poulard et al., 2015). The CORA can evaluate the similarity between curves by the cross correlation method, which evaluates error according to phase shift, magnitude and curve shape to produce a relative score ranging from 0 (no correlation) to 1 (perfect match) (Miller et al., 2017; Decker et al., 2020).

## Results

The simulation results of component level validations were presented in the Supplementary Figures 3, 7, except the results of FSU flexion bending testing and rear hub impact for T-spine. The CORA ratings were collected in the Table 1.

**TABLE 1:** CORA scores for different validation cases.

Body region(s)	Load cases	Responses/signals	CORA score
Thoracic intervertebral joints	Markolf (1972)	Tension	0.88
		Compression	0.92
		Shearing	0.90
		Lateral bending	0.70
	Lopez-Valdes et al. (2011)	T234 (M-θ)	0.79
		T789 (M-θ)	0.83
	Forman et al. (2015)	Chest deflection	0.94
		Change in spine angle	0.89
Costovertebral joints	Duprey et al. (2010)	R2 ventral	0.92
		R2 dorsal	0.67
		R2 cranial	0.78
		R2 caudal	0.17
		R4 ventral	0.84
		R4 dorsal	0.69
		R4 cranial	0.77
		R4 caudal	0.64
		R6 ventral	0.84
		R6 dorsal	0.69
		R6 cranial	0.71
		R6 caudal	0.72
		R8 ventral	0.76
		R8 dorsal	0.77
		R8 cranial	0.78
		R8 caudal	0.73
		R10 ventral	0.68
		R10 dorsal	0.68
		R10 cranial	0.67
		R10 caudal	0.72
Clavicle	Zhang et al. (2014)	Force-defl (three-point bending)	0.87
		Force-defl (axial compression)	0.82
		Strain-force (three-point bending)	0.90
		Strain-force (axial compression)	0.89
Sternum	Kerrigan et al. (2010)	Force-time (proximal end)	0.62
		Force-time (distal end)	0.53
		Rotation angle-time (proximal end)	0.94
		Rotation angle-time (distal end)	0.90
Costal-cartilage injury	Forman et al. (2010)	Force-disp (w. perichondrium)	N/A
		Force-disp (w/o perichondrium)	N/A
Eviscerated ribcage: point loading	Kindig et al. (2010)	Upper sternum	0.81
		Lower sternum	0.84
		Rib1_CCJ	0.86
		Rib3_CCJ	0.85
		Rib4_CCJ	0.91
		Rib6_CCJ	0.84
FBM: frontal pendulum impact	Kroell et al. (1971)	Deflection-time	0.98
		Force-time	0.96
FBM: shoulder pendulum impact	Koh et al. (2005)	D-t (4.4 m/s padded)	0.78
		F-t (4.4 m/s padded)	0.76
		D-t (4.5 m/s unpadded)	0.87
		F-t (4.5 m/s unpadded)	0.88
		D-t (6.4 m/s padded)	0.99
		F-t (6.4 m/s padded)	0.91
		D-t (6.8 m/s unpadded)	0.95
		F-t (6.8 m/s unpadded)	0.86
FBM: table top tests	Kent et al. (2004)	Force-compression (single belt)	0.92
		Force-compression (double belts)	0.80
		Force-compression (hub loading)	0.88
		Force-compression (distributed loading)	0.90

### Thoracic Intervertebral Joints

**FIGURE 9:**
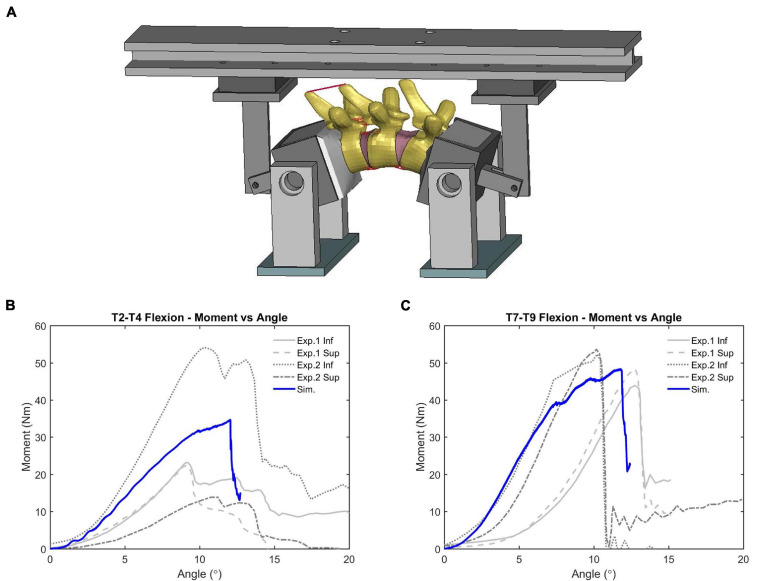
Flexion bending validation results of FSU: **(A)** intervertebral ligaments rupture (FSU T7-T9), and **(B,C)** showed the moment-angle response obtained from FSU T2-T4 and T7-T9.

The simulation results of adjacent vertebrae to quasi-static external loads (force vs. disp., or moment vs. rotation) produced reasonable responses (see Supplementary Figure 3) compared to experimental data (Markolf, 1972). The average CORA score was 0.85, showing good correlation with the testing data. For dynamic flexion testing of upper-thoracic and mid-thoracic FSU, ligament rupture was observed in the deformed configuration of the updated model, e.g., ligaments between T8 and T9 (Figure 9A). The moment-angle curve generated by our updated model exhibited the toe-region followed by increasing stiffness that is characteristic of FSU joint response (Figures 9B,C). A region with sub-traumatic damage appeared until the peak moment before a significant drop in response, which matched the experimental results. The simulated peak moment before failure was 31.8 Nm for FSU T2-T4 and 48.3 N⋅m for FSU T7-T9, consistent with the experimental data. The average CORA score was 0.81, showing an overall reasonable correlation with the limited testing data. These results in the flexion simulation showed good agreement with soft tissue injuries and failure moment described in the observed experimental testing.

**FIGURE 10:**
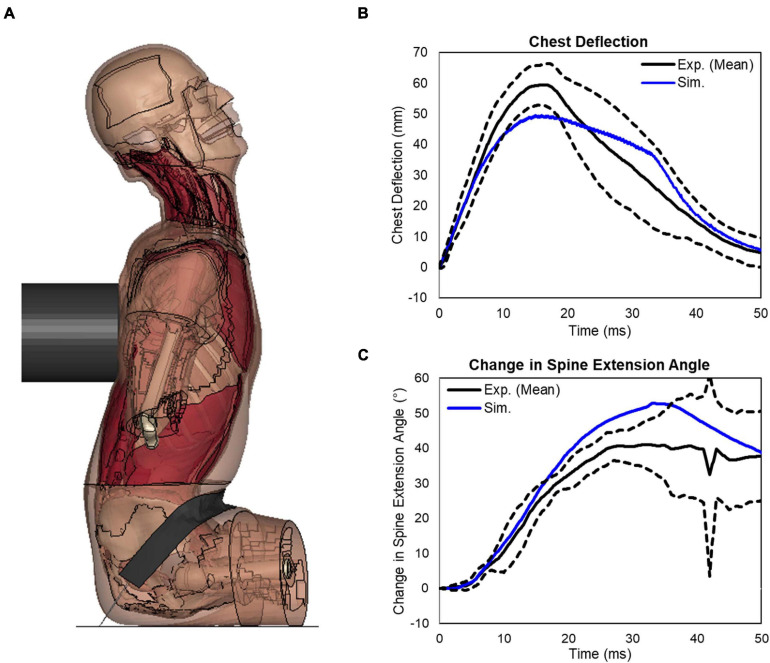
Extension bending of spine under rear blunt impact on full torso: **(A)** a deformed configuration of the model under the blunt rear impact, **(B)** response of the chest deflection, and **(C)** response of the change in spine extension angle.

Following the FSU validation, a validation case involving a hub impact to the spine was simulated and a deformed configuration was shown in Figure 10A. The impact loading to the T-spine resulted in gross chest compression and forced the T-spine into extension. For all three impact speed tests, the impact force response produced by the model fell within the upper and lower bounds of the experimental data (Forman et al., 2015). For the 5.5 m/s impact, the peak chest deflection was close to the lower bound of the experimental data (Figure 10B). For the spine extension angle, the simulation was close to the upper bound of the experiment data (Figure 10C). Quantitatively, the CORA score for chest deflection was 0.94, and for the change in spine angle it was 0.89, showing a very good correlation with the testing data.

### Costovertebral Joints

The results for all simulated costovertebral joints under each loading direction were summarized and provided in Supplementary Figure 4. Each of the cases had 2 to 3 moment-angle curves from experimentation associated with it, and often there was considerable variation in the apparent stiffness. Nevertheless, the CORA scores of most of the simulation results was 0.7 or greater, and the response was within the range of the experiments, except the ribs 2 under caudal motion. There were also some discrepancies for dorsal motion, which often produced stiffer responses likely due to contacts between the ribs and the transverse processes. It is unknown whether a similar effect was present in Duprey’s tests (Duprey et al., 2010).

### Clavicle and Sternal Modeling

The experiment testing reported reaction forces (along the direction of loading) against the deflection, and the peak bone surface strain against the forces. To evaluate the clavicle response, the simulation results were compared to the response obtained from three-point bending tests and axial compression tests, including the force versus the deflection and the peak cortical bone strain versus the force. It was noted that the response of the base model was softer when compared to the testing data. After a parametric study of the material properties, the elastic modulus of cortical clavicle bone was updated from 9 to 18 GPa, and the yield strength was increased from 0.08 to 0.16 GPa. The force-deflection response and peak strain-force response obtained from updated cortical bone model were plotted, which were reasonable compared to testing data (see Supplementary Figure 5).

For the three-point bending test of the sternum, the vertical reaction force was measured from the two supporting posts, which were associated with the proximal and distal ends of the sternum. The vertical force response obtained from the base model showed much higher peak force than the experiments. After performing parametric studies of material properties on cortical bone, the cortical sternum bone modulus was reduced from 14 to 4 GPa, and reducing yield strength from 0.09 to 0.035 GPa. The 2% MPS threshold was adopted to represent the strain based failure feature of cortical sternum bone. The vertical reaction force response on each post was plotted and compared with the experimental data (see Supplementary Figure 6). The peak force and the slope of the force-time curve matched the range of the testing data well.

### Costal Cartilage Modeling

The experiment reported force-displacement results of the paired tests: perichondrium-intact and perichondrium-removed tests for each specimen. The peak forces (along anteroposterior direction) from each of the perichondrium-removed tests were normalized by dividing them by the peak forces of the matching perichondrium-intact tests. The simulation results of the model with optimized material parameters (the elastic modulus *E* = 12.5 MPa for the solid cartilage and *E* = 55 MPa for the fabric perichondrium shell layer) were compared to the results of the experimental data (see Supplementary Figure 7). Compared to the FE model with perichondrium layer, the peak stress obtained from the model without perichondrium decreased about 45%, which was very similar to the 47% for the average ratio of measured peak force from the experiment.

### Point Loading of the Eviscerated Ribcage

**FIGURE 11:**
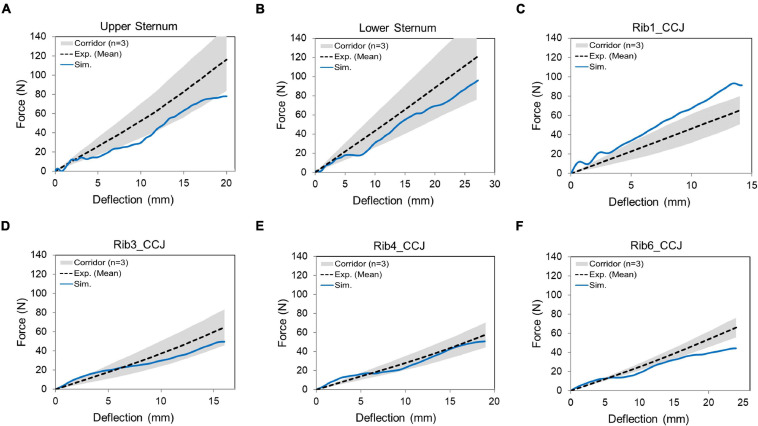
Force-deflection response for the denuded ribcage under point loading on different loading sites: **(A)** upper sternum, **(B)** lower sternum, and **(C–F)** displayed costochondral junction (CCJ) of the rib levels 1, 3, 4, and 6.

The predicted force-deflection responses from the ribcage model were compared with the experimental data (Figure 11). The rib 1 was overly stiff to a certain extent (i.e., the force was about 14% larger than the upper limit of the corridor when the maximum deflection was reached), and the rib 6 was close to the lower bound of the corridor. The CORA scores ranged from 0.81 to 0.91. Overall, the model responses agreed with the experimental corridors, which indicated that the ribcage stiffness was comparable to the tested three subjects.

### Frontal Pendulum Impact

**FIGURE 12:**
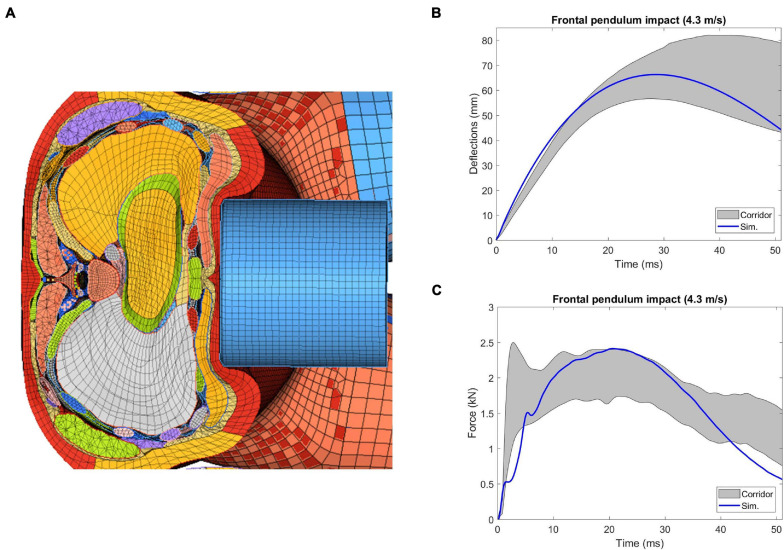
Simulation results of thorax model under Kroell et al. (1971) frontal pendulum impact: **(A)** superior view of the cross-section of the compressed thorax at the mid-sternum, **(B)** deflection-time response, and **(C)** force-time response.

The sectional view of the thorax configuration under the frontal pendulum impact (25 ms) was provided in Figure 12A, which showed the ribcage and internal organs were compressed along the anteroposterior direction. The deflection and force responses predicted by the model were mostly within the range of the experimental corridors (Figures 12B,C). Quantitatively, the CORA score for chest deflection was 0.98, and for force response was 0.90, showing a very good correlation with the testing results.

### Shoulder Pendulum Impact

**FIGURE 13:**
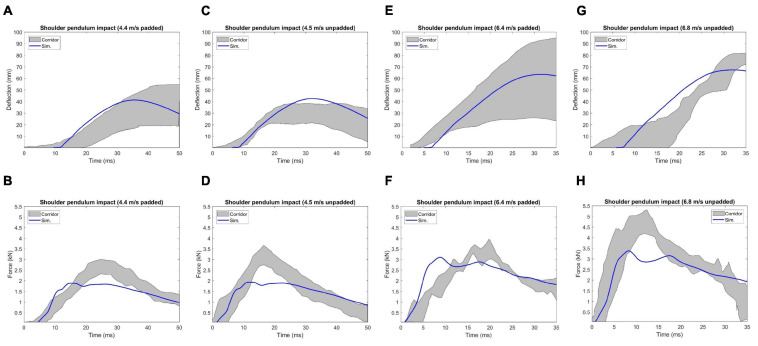
Deflection and force responses under Koh et al. (2005) shoulder pendulum impact: **(A)** and **(B)** 4.4 m/s padded impact, **(C,D)** 4.5 m/s unpadded impact, **(E,F)** 6.4 m/s padded impact, and **(G,H)** 6.8 m/s unpadded impact.

In Figure 13, the model responses were compared with the experimental corridors, which were developed by Koh et al. (2005) based on biomechanical responses from four cadaveric studies. The overall deflection responses under different loading conditions were reasonable compared to the corridors, except for the 6.8 m/s unpadded impact, where the peak deflection predicted by the model was smaller to some extent compared to the range of the corridors. Compared to the corridor under each loading condition, the simulation predicted peak force appeared earlier and the value was lower than the corridor. The average CORA score of the chest deflection for the four loading cases was 0.90, and it was 0.85 for force response.

### Table-Top Restraint-Like Loading Tests

**FIGURE 14:**
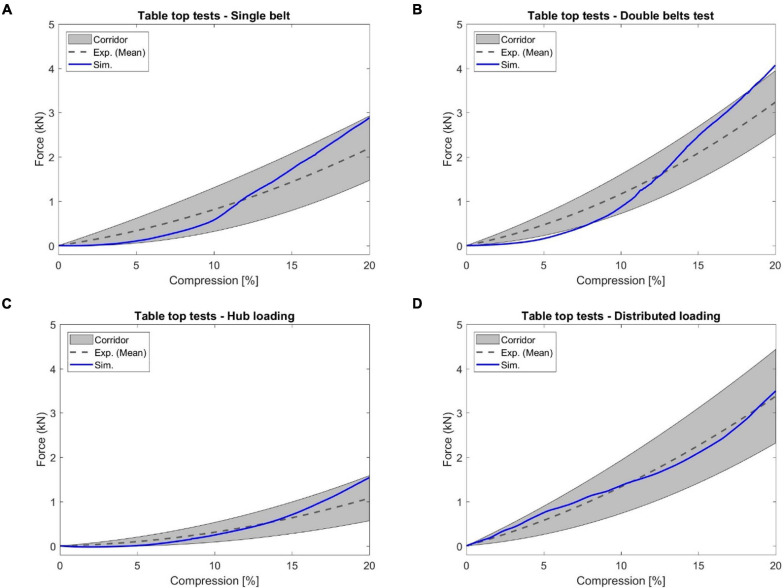
The reaction force versus chest compression responses produced by the table top tests: **(A)** single diagonal belt loading, **(B)** double diagonal belts loading, **(C)** hub loading, and **(D)** distributed loading.

In Figure 14, the simulated reaction force versus chest compression responses agreed well with the corresponding experimental corridors (Kent et al., 2004). The response curve produced by the double diagonal belts loading was slightly outside of the corridor bounds during some chest compression rates (e.g., less than 7% compression). However, the response curve produced by each of the other three loading conditions was within the corridor range. Quantitatively, the CORA ranged from 0.80 to 0.90, which indicated good correlation between simulation and testing results.

## Discussion

In this study, a hierarchical process was presented to evaluate, validate, and improve the musculoskeletal modeling of a mid-sized male thorax for impact scenarios. At the component level, the modeling enhancement and improvement for major bony structures of the thorax was introduced, including the thoracic intervertebral joints, costovertebral joints, clavicle, sternum and costal cartilages. At ribcage and full thorax levels, the thorax model with validated components was quantitatively evaluated by four types of experimental testing.

### Body Parts Validation and Improvement

Most published thoracic spine models for impact analysis were part of thoracic or whole body models with oversimplified model features (Yang, 2018). For example, the GHBMC M50 v4.5 model (version released in 2016) used drum-like closed surfaces to represent intervertebral discs, and the spinous ligaments were not included in the model. In this work, the ligaments were modeled using available material property data from available literature, and the annulus fibrosus fiber lamina layers were created and embedded into the solid discs. Three levels of independent experimental tests have been conducted for validating the response of our enhanced T-spine model: adjacent vertebrae/segment, FSU, and full torso. For adjacent vertebrae in response to quasi-static loads (Markolf, 1972), the results of force vs. displacement and moment vs. rotation of T8-T9 showed good agreement with the experimental results. However, it should be noted that the magnitudes of deformation or rotation under these quasi-static loads were very small (e.g., the maximum displacement in the reported shear experiments was less than 0.8 mm), which means slight variations between model setup and experiment were likely to cause discrepancies. The FSU flexion bending testing applied loading until soft tissue failure, which could describe the injury behavior of the upper and mid-thoracic spine at the segmental level. The initial toe region of the moment response and ligament rupture were well reproduced in the results (Figure 9) of our enhanced T-spine model with realistic IVD and non-linear ligaments. It was noted that the experimental study only included two specimens, which produced a wide range of response curves and failure moments. The blunt impacts on the back of thorax validated the chest deflection and spine extension angle of model, which showed the overall spine kinematic response under rear impact agreed well with the experimental results. For non-injurious impact testing cases (1.5 and 3.0 m/s), although the responses of deflection and change of spine extension angle were not presented here, they showed good agreement with the experimental results. The 5.5 m/s impact velocity was designed for potentially injurious situation. However, it lacked spinal injury in the experiment for the first two subjects with a nominal stroke distance of 85 mm. The impact stroke was increased to 150 mm for the final two subjects. Our simulation was conducted using 150 mm stroke distance. This explains why the simulated spine extension angle was at the upper limit of the data based on four subjects.

The simulation of the costovertebral joints under quasi-static ventral-dorsal flexion and cranial-caudal flexion predicted fair to good results compared to Duprey et al. (2010) test data, except the moment-angle produced by rib 2 caudal flexion. This could be attributed to the limited sample and often the samples themselves having wide range of response. Furthermore, the process of tuning the material properties of the ligaments associated with the costovertebral joint was complicated by the fact that many of the beams in the model, whether they represent the costotransverse, superior costotransverse, or lateral costotransverse ligaments, all couple and contribute to the moment-angle relationship for the 4 simulated directions. Stiffening up the beams to produce a better caudal response appears to also cause substantial deviations in the cranial, ventral, and dorsal relations. In a larger scope, multiple output responses of a computational model could be sensitive to the changes of a specific input parameter. it is important to ensure proper characterization of such parameters that have a significant impact on model outputs.

For clavicle and sternal validation and improvement, the material parameters were determined based on parametric studies of material properties. The adjusted material properties can compensate for missing model details or geometry deficiencies (Cronin, 2014), aiming to achieve better prediction of global kinematic response and component level injury. For clavicle under three-point bending and axial compression (Zhang et al., 2014), the predicted responses were within the range of experimental curves and the CORA scores were good, although the strain vs. force response was close to the upper bound of experimental data. Qualitatively, the trends of the simulated results of the three-point bending of sternum agreed well with the testing data (Kerrigan et al., 2010). However, the model only showed fair agreement with the experimental results according to the quantitative CORA scores, probably due to the large variability observed in testing data. To enhance the costal cartilage model, a layer of perichondrium with a fabric material was created to surround the solid mesh of the costal cartilage. Biomechanically, the perichondrium has been shown to contribute approximately half of the force response under cantilever-beam like bending (Forman et al., 2010), when comparing the normalized force-displacement curves produced by perichondrium-intact model and perichondrium-removed model. A similar distribution of reaction forces was achieved by tuning the Young’s moduli for the costal cartilage and perichondrium. The results were within the testing range and reported experimental error.

### Ribcage and Full Thorax Levels Validation

Once the presented body components were enhanced by adding more biofidelic details or adjusting material properties, and validated by adopted experimental loading cases, these components were integrated into the thorax in the FBM. To check the stability and biomechanical responses of thorax model, validations were conducted under a wide variety of loading scenarios at ribcage or full thorax level.

In this study, the validated sternum, costal cartilages, and thoracic spine were integrated into the thorax model. The geometry and material properties of the ribs in the current thorax model were not changed since they were already validated in previous studies (Li et al., 2010; Poulard et al., 2015; Guleyupoglu et al., 2017; Zaseck et al., 2018). The quasi-static point loading of the eviscerated ribcage (Kindig et al., 2010) was utilized in this work to evaluate the ribcage model response with updated compoments. It should be noted that experimental corridors were developed based on only three denuded and eviscerated PMHS. The simulated force vs. deflection curves followed the expected trends established in the corridors, and were lower than the average experimental results, except in the case with point loading on the costochondral junction of rib level 1 (Rib1_CCJ). The frontal pendulum impact (Kroell et al., 1971) was simulated to evaluate the global chest response along the anteroposterior direction, including the chest deflection and impact force. The shoulder pendulum impact (Koh et al., 2005) was modeled to evaluate the overall response of the upper thorax along the lateral direction under four loading speed cases, including the deflection and impact force. Most of these pendulum impact cases were generally well correlated with the corresponding experimental validation corridors, except some local regions. This could be attributed to the modeling deficiencies of the shoulders (Xu et al., 2018), which lacked credible experimental testing data for validation thus far. Also, some details regarding how the corridors were produced were unknown, which could be a source of discrepancy between simulation and testing corridors. The predicted chest force vs. compression responses under the table-top restraint-like loading tests (Kent et al., 2004) were within the upper and lower bounds of testing data. Quantitatively, the average CORA score was 0.88, which indicated the model responses closely agreed with the corridors.

In summary, to develop a credible thorax FE model, there is a need for a computational approach that can evaluate and validate model response for the sake of achieving high biofidelity. This work presented a hierarchical approach to validate and enhance thorax model biofidelity. Based on relevance and necessity to improve the current GHBMC thorax model, as well as the availability of experimental data, the aforementioned test cases were selected for validation at both component and whole thorax or torso levels. Overall, the model responses showed good or close agreement with the experimental data both qualitatively and quantitatively.

### Limitations of This Study

Detailed human thorax body models can provide insights to investigate injury mechanisms and tolerances by qualitative assessment and quantitative evaluation of thoracic response at tissue level. However, similar to developing other human body models, there are some limitations of this work which may or may not affect the model biofidelity. First, there were a paucity of available experimental data or missing technical details regarding specific aspects of model verifications or validations. Unfortunately, most experimental data were not developed or presented for the purposes of computational modeling studies (Yang, 2018). Also, some experimental tests utilized for our model validation were conducted more than three or four decades ago. Some details related to the testing subjects or test conditions were missing or lacked adequate description. For instance, the frontal pendulum impact on chest (Kroell et al., 1971) were published in the 1970s.

Second, there were some model simplifications due to insufficient representation of anatomical details or material properties. For example, all ligaments related to the thoracic intervertebral joints and costovertebral joints were modeled using 1D elements, rather than a faithful representation of the 3D geometry. Also, the regional differences in cortical bone thickness were not considered in this thorax model, except for the ribs.

Third, the model presented in the current study was not able to accurately predict local strains of the thoracic skeleton. At the component level validation, the strain-force response of the clavicle was compared between simulation and experimental results, since the data was available. However, reliable experimental data is needed to evaluate the strain distribution elsewhere within the whole ribcage. Even though a model can be validated against global impact responses, the local strains of most parts at tissue level are not able to be validated since cortical strain was relatively insensitive to the model inputs (Anderson et al., 2005) in a full body loading environment. While it was noted that the rib modeling and fractures of this presented thorax model has been validated in previous studies, a newer probabilistic method may be a better computational strategy in future investigations to predict rib fracture risk within FBM (Forman et al., 2012).

Finally, the current model was created based on a single mid-sized subject, which is not a real population-averaged model. The medical images for CAD data of this model were collected from a young (26-year-old) living male volunteer (Gayzik et al., 2011). Age-related factors affecting the thorax modeling such as morphologic and material characteristics were not investigated. The human ribcage morphology changes as it ages through adulthood. Also, due to the increased porosity and decreased mineralization associated with aging, the thinning of the cortical shell and changing bone material properties could change the biomechanics and injury tolerance of the thorax (Kent et al., 2005). For example, the tolerable sternal deflection level is much lower in the aging bony thorax. It is worth emphasizing that the modeling and validation work presented in this study were focused on biofidelity and capability of injury prediction, and further research is needed to validate the model for other applications (e.g., orthopedic biomechanics).

## Conclusion

This work presented a comprehensive evaluation and validation procedure to hierarchically improve and enhance the MSK system of a mid-sized male thorax FE model. The material characteristics of relevant thoracic bony structures and ligaments were either improved by parametric study or determined based on available literature. To achieve high biofidelity, new model features were developed and incorporated into the chest components, such as thoracic intervertebral joints and costal cartilage. For the sake of accurately predicting injury risk at both component and whole thorax levels, a multi-level validation process was presented in this study to maximize the capability of FE model-predicted impact responses. The whole ribcage or thorax model was evaluated against a wide range of loads and different modes of loading. Compared with those corresponding experimental data sets, the CORA ratings of the model ranged from 0.76 to 0.99, indicating the model provides good predictions of the overall biomechanical responses of the whole thorax. This validated, highly biofidelic thorax model has been integrated into the newest generation of GHBMC models to represent the state-of-the-art computational human body models for crash injury prediction and prevention. Future studies will focus on tissue-level injury predictions based on reliable experimental data, as well as extending the framework of accurate modeling and multi-level validation procedure for different population groups including age- and gender-based computational models.

## Data Availability Statement

The raw data supporting the conclusions of this article will be made available by the authors, without undue reservation.

## Author Contributions

All authors contributed to the conception, design, and interpretation of results presented in this study, and are accountable for all aspects of this work.

## Conflict of Interest

The authors declare that the research was conducted in the absence of any commercial or financial relationships that could be construed as a potential conflict of interest.

## References

[B1] Aira J. Guleyupoglu B. Jones D. Koya B. Davis M. Gayzik F. S. (2019). Validated thoracic vertebrae and costovertebral joints increase biofidelity of a human body model in hub impacts. *Traffic Inj. Prev.* 20 S1–S6. 10.1080/15389588.2019.1638511 31364878

[B2] Anderson A. E. Peters C. L. Tuttle B. D. Weiss J. A. (2005). Subject-specific finite element model of the pelvis: development, validation and sensitivity studies. *J Biomech. Eng.* 127 364–373. 10.1115/1.1894148 16060343

[B3] Antona-Makoshi J. Yamamoto Y. Kato R. Sato F. Ejima S. Dokko Y. (2015). Age-dependent factors affecting thoracic response: a finite element study focused on Japanese elderly occupants. *Traffic Inj. Prev.* 16 S66–S74. 10.1080/15389588.2015.1014552 26027977

[B4] Beeman S. M. Kemper A. R. Madigan M. L. Franck C. T. Loftus S. C. (2012). Occupant kinematics in low-speed frontal sled tests: Human volunteers. *Hybrid III ATD, and PMHS. Accid. Anal. Prev.* 47 128–139. 10.1016/j.aap.2012.01.016 22342960

[B5] Cavanaugh J. H. Yoganandan N. (2015). “Thoracic injury biomechanics,” in *Accidental Injury: Biomechanics and Prevention, Third ed*, eds Yoganandan N. Nahum A. M. Melvin J. W. (Berlin: Springer), 331–372.

[B6] Chazal J. Tanguy A. Bourges M. Gaurel G. Escande G. Guillot M. (1985). Biomechanical properties of spinal ligaments and a histological study of the supraspinal ligament in traction. *J. Biomech.* 18 167–176. 10.1016/0021-9290(85)90202-7 3997901

[B7] Cronin D. S. (2014). Finite element modeling of potential cervical spine pain sources inneutral position low speed rear impact. *J. Mech. Behav. Biomed. Mater.* 33 55–66. 10.1016/j.jmbbm.2013.01.006 23466282

[B8] Decker W. B. Baker A. M. Ye X. Brown P. J. Stitzel J. D. Gayzik F. S. (2020). Development and multi-scale validation of a finite element football helmet model. *Ann. Biomed. Eng.* 48 258–270. 10.1007/s10439-019-02345-7 31520331 PMC6928099

[B9] Deng Y.-C. Kong W. Ho H. (1999). *Development of a finite element human thorax model for impact injury studies. SAE Technical Paper, 1999–01–0715.* Pennsylvania: SAE. 10.4271/1999-01-0715

[B10] Duprey S. Bruyere K. Veriest J. (2008). Influence of geometrical personalization on the simulation of clavicle fractures. *J. Biomech.* 41 200–207. 10.1016/j.jbiomech.2007.06.020 17697683

[B11] Duprey S. Subit D. Guillemot H. Kent R. W. (2010). Biomechanical properties of the costovertebral joint. *Med. Eng. Phys.* 32 222–227. 10.1016/j.medengphy.2009.12.001 20036178

[B12] El-Jawahri R. E. Laituri T. R. Ruan J. S. Rouhana S. W. Barbat S. D. (2010). Development and validation of age-dependent FE human models of a mid-sized male thorax. *Stapp Car Crash J.* 54 407–430. 21512916 10.4271/2010-22-0017

[B13] Forman J. Perry B. Henderson K. Gjolaj J. P. Heltzel S. Lessley D. (2015). Blunt impacts to the back: Biomechanical response for model development. *J. Biomech.* 48 3219–3226. 10.1016/j.jbiomech.2015.06.035 26184586

[B14] Forman J. Poplin G. S. Shaw C. G. McMurry T. L. Schmidt K. Ash J. (2019). Automobile injury trends in the contemporary fleet: Belted occupants in frontal collisions. *Traffic Inj. Prev.* 20 607–612. 10.1080/15389588.2019.1630825 31283362

[B15] Forman J. L. del Pozo de Dios E. Dalmases C. A. Kent R. W. (2010). The contribution of the perichondrium to the structural mechanical behavior of the costal-cartilage. *J. Biomech. Eng.* 132 094501. 10.1115/1.4001976 20815649

[B16] Forman J. L. Kent R. W. Mroz K. Pipkorn B. Bostrom O. Segui-Gomez M. (2012). “Predicting rib fracture risk with whole-body finite element models: development and preliminary evaluation of a probabilistic analytical framework,” in *Proceedings of The 56th Annual AAAM Scientific Conference*, (Seattle, WA), 109. PMC3503420 23169122

[B17] Gayzik F. S. Hamilton C. A. Tan J. C. McNally C. Duma S. M. Klinich K. D. (2009). *A multi-modality image data collection protocol for full body finite element model development. SAE Technical Paper, 2009-01-2261.* Pennsylvania: SAE. 10.4271/2009-01-2261

[B18] Gayzik F. S. Moreno D. P. Geer C. P. Wuertzer S. D. Martin R. S. Stitzel J. D. (2011). Development of a full body CAD dataset for computational modeling: a multi-modality approach. *Ann. Biomed. Eng.* 39 2568–2583. 10.1007/s10439-011-0359-5 21785882

[B19] Gehre C. Gades H. Wernicke P. (2009). “Objective rating of signals using test and simulation responses,” in *Paper Presented at: 21st International Technical Conference on the Enhanced Safety of Vehicles Conference (ESV)*, (Stuttgart, Germany).

[B20] Guleyupoglu B. Barnard R. Gayzik F. S. (2017). *Automating regional rib fracture evaluation in the GHBMC detailed average seated male occupant model. SAE Technical Paper, 2017-01-1428.* Pennsylvania: SAE. 10.4271/2017-01-1428

[B21] Haug E. Choi H.-Y. Robin S. Beaugonin M. (2004). “Human models for crash and impact simulation,” in *In: Handbook of Numerical Analysis*, ed. Ciarlet P. G. (Amsterdam: Elsevier), 231–452. 10.1016/s1570-8659(03)12004-2

[B22] Hill R. (1979). Aspects of invariance in solid mechanics. *Adv. Appl. Mech.* 18 1–75. 10.1016/S0065-2156(08)70264-3

[B23] Iwamoto M. Nakahira Y. Kimpara H. (2015). Development and validation of the total human model for safety (THUMS) toward further understanding of occupant injury mechanisms in precrash and during crash. *Traffic Inj. Prev.* 16 (Suppl. 1), S36–S48. 10.1080/15389588.2015.1015000 26027974

[B24] Kent R. Lee S. H. Darvish K. Wang S. Poster C. S. Lange A. W. (2005). Structural and material changes in the aging thorax and their role in crash protection for older occupants. *Stapp Car Crash J.* 49 231–249. 17096276 10.4271/2005-22-0011

[B25] Kent R. Lessley D. Sherwood C. (2004). Thoracic response to dynamic, non-impact loading from a hub, distributed belt, diagonal belt, and double diagonal belts. *Stapp Car Crash J.* 48, 495–519. 17230280 10.4271/2004-22-0022

[B26] Kerrigan J. R. Bose D. Li Z. Arregui-Dalmases C. Pozo E. D. (2010). Response of the sternum under dynamic 3-point bending. *Biomed. Sci. Instrum.* 46 440–445. 20467120

[B27] Kimpara H. Lee J. B. Yang K. H. King A. I. (2005). Development of a three-dimensional finite element chest model for the 5th percentile female. *Stapp Car Crash J.* 49 251–269. 17096277 10.4271/2005-22-0012

[B28] Kindig M. Li Z. Kent R. Subit D. (2015). Effect of intercostal muscle and costovertebral joint material properties on human ribcage stiffness and kinematics. *Comput. Methods Biomech. Biomed. Eng.* 18 556–570. 10.1080/10255842.2013.820718 23947597

[B29] Kindig M. W. Lau A. G. Forman J. L. Kent R. W. (2010). Structural response of cadaveric ribcages under a localized loading: stiffness and kinematic trends. *Stapp Car Crash J.* 54 337–380. 21512914 10.4271/2010-22-0015

[B30] Koh S. W. Cavanaugh J. M. Mason M. J. Petersen S. A. Marth D. R. Rouhana S. W. (2005). Shoulder injury and response due to lateral glenohumeral joint impact: an analysis of combined data. *Stapp Car Crash J*. 49, 291–322. 17096279 10.4271/2005-22-0014

[B31] Kroell C. Schneider D. Nahum A. (1971). *Impact tolerance and response of the human thorax. SAE Technical Paper, 710851.* Pennsylvania: SAE. 10.4271/710851

[B32] Lebarbe M. Petit P. (2012). “New biofidelity targets for the thorax of a 50th percentile adult male in frontal impact,” in *Paper Presented at: International IRCOBI Conference* (Dublin, Ireland).

[B33] Li Z. Kindig M. W. Subit D. Kent R. W. (2010). Influence of mesh density, cortical thickness and material properties on human rib fracture prediction. *Med. Eng. Phys.* 32 998–1008. 10.1016/j.medengphy.2010.06.015 20674456

[B34] Lopez-Valdes F. J. Lau S. Riley P. Lamp J. Kent R. (2011). The biomechanics of the pediatric and adult human thoracic spine. *Ann. Adv. Automot. Med.* 55 193–206. 22105396 PMC3256823

[B35] Markolf K. L. (1972). Deformation of the thoracolumbar intervertebral joints in response to external loads: a biomechanical study using autopsy material. *J. Bone Joint Surg. Am.* 54 511–533. 10.2106/00004623-197254030-00005 5055150

[B36] Miller L. E. Urban J. E. Stitzel J. D. (2017). Validation performance comparison for finite element models of the human brain. *Comput Methods Biomech Biomed Engin.* 20 1273–1288. 10.1080/10255842.2017.1340462 28701050 PMC5975353

[B37] Newell N. Little J. P. Christou A. Adams M. A. Adam C. J. Masouros S. D. (2017). Biomechanics of the human intervertebral disc: a review of testing techniques and results. *J. Mech. Behav. Biomed. Mater* 69 420–434. 10.1016/j.jmbbm.2017.01.037 28262607

[B38] Panzer M. B. Cronin D. S. (2009). C4–C5 segment finite element model development, validation, and load-sharing investigation. *J. Biomech.* 42 480–490. 10.1016/j.jbiomech.2008.11.036 19200548

[B39] Panzer M. B. Fice J. B. Cronin D. S. (2011). Cervical spine response in frontal crash. *Med. Eng. Phys.* 33 1147–1159. 10.1016/j.medengphy.2011.05.004 21665513

[B40] Pintar F. A. (1986). *The biomechanics of spinal elements. PhD Dissertation.* Milwaukee: Marquette University.

[B41] Pipkorn B. Kent R. (2011). “Validation of a human body thorax model and its use for force, energy and strain analysis in various loading conditions,” in *Paper Presented at: International IRCOBI Conference*, (Krakow, Poland).

[B42] Poulard D. Kent R. W. Kindig M. Li Z. Subit D. (2015). Thoracic response targets for a computational model: A hierarchical approach to assess the biofidelity of a 50th-percentile occupant male finite element model. *J. Mech. Behav. Biomed. Mater.* 45 45–64. 10.1016/j.jmbbm.2015.01.017 25681717

[B43] Robin S. (2001). “Human Model for Safety – a joint effort towards the development of redefined human-like car-occupant models,” in *Proceedings of the 17th International Conference for the Enhanced Safety of Vehicles*, (Amsterdam, Netherlands).

[B44] Schoell S. L. Weaver A. A. Vavalle N. A. Stitzel J. D. (2015). Age-and sex-specific thorax finite element model development and simulation. *Traffic Inj. Prev.* 16 (Suppl. 1), S57–S65. 10.1080/15389588.2015.1005208 26027976

[B45] Shen W. Niu Y. Mattrey R. F. Fournier A. Corbeil J. Kono Y. (2008). Development and validation of subject-specific finite element models for blunt trauma study. *J. Biomech. Eng.* 130 021022. 10.1115/1.2898723 18412509

[B46] Song E. Trosseille X. Baudrit P. (2009). Evaluation of thoracic deflection as an injury criterion for side impact using a finite elements thorax model. *Stapp Car Crash J.* 53 155–191. 20058554 10.4271/2009-22-0006

[B47] Vavalle N. A. Jelen B. C. Moreno D. P. Stitzel J. D. Gayzik F. S. (2013). An evaluation of objective rating methods for full-body finite element model comparison to PMHS tests. *Traffic Inj. Prev* 14 S87–S94. 10.1080/15389588.2013.802777 23905846

[B48] Xu T. Sheng X. Zhang T. Liu H. Liang X. Ding A. (2018). Development and validation of dummies and human models used in crash test. *Appl. Bionics. Biomech.* 2018 3832850. 10.1155/2018/3832850 30538770 PMC6257900

[B49] Yang K. H. (2018). *Basic finite element method as applied to injury biomechanics.* Cambridge: Academic Press.

[B50] Yang K. H. Hu J. White N. A. King A. I. Chou C. C. Prasad P. (2006). Development of numerical models for injury biomechanics research: a review of 50 years of publications in the Stapp Car Crash Conference. *Stapp Car Crash J.* 50 429–490. 17311173 10.4271/2006-22-0017

[B51] Zaseck L. W. Chen C. Hu J. Reed M. P. Rupp J. (2018). The influence of pre-existing rib fractures on Global Human Body Models Consortium thorax response in frontal and oblique impact. *J. Biomech.* 69 54–63. 10.1016/j.jbiomech.2018.01.010 29373114

[B52] Zhang Q. Kindig M. Li Z. Crandall J. R. Kerrigan J. R. (2014). Development of structural and material clavicle response corridors under axial compression and three point bending loading for clavicle finite element model validation. *J. Biomech.* 47 2563–2570. 10.1016/j.jbiomech.2014.06.004 24975696

[B53] Zhu F. Jiang B. Hu J. Wang Y. Shen M. Yang K. H. (2016). Computational modeling of traffic related thoracic injury of a 10-year-old child using subject-specific modeling technique. *Ann. Biomed. Eng.* 44 258–271. 10.1007/s10439-015-1372-x 26126484

