# Supplementary material for: Evaluation and Validation of Thorax Model Responses: A Hierarchical Approach to Achieve High Biofidelity for Thoracic Musculoskeletal System
Source: Front Bioeng Biotechnol. 2021 Jul 16;9:712656. doi: 10.3389/fbioe.2021.712656 (PMC8324103; doi:10.3389/fbioe.2021.712656)
Supplement: Supplementary file 1 [file Data_Sheet_1.docx]

**Supplementary Materials**

**SM-A: The material models and properties for some important parts in the model.**

Table 1. Material models and properties for the important components relevant to the model in current study.

| **Part/Tissue** | | **Element type** | **Constitutive model** | **Material parameters** |
| --- | --- | --- | --- | --- |
| Clavicle (cortical) | | Shell | Piecewise linear plasticity | *E*=18 GPa, *σ*_Y_=0.16, *E*_t_=0.9 GPa |
| Clavicle (trabecular) | | Solid | Piecewise linear plasticity | *E*=0.5 GPa, *σ*_Y_=0.0018, *E*_t_=0.001 GPa |
| Sternum (cortical) | | Shell | Piecewise linear plasticity | *E*=4 GPa, *σ*_Y_=0.035, *E*_t_=2.3 GPa |
| Sternum (trabecular) | | Solid | Piecewise linear plasticity | *E*=0.04 GPa, *σ*_Y_=0.0022, *E*_t_=0.001 GPa |
| Costal cartilage | | Solid | Piecewise linear plasticity | *E*=0.0125 GPa, *σ*_Y_=0.0015, *E*_t_=0.00125 GPa |
| Perichondrium | | Shell | Fabric | *E*_a_=0.055 GPa |
| Nucleus pulposus | | Solid | Elastic fluid | *K*=1.72 GPa |
| Annulus fibrosus | | Solid | Hill foam | *K*=1 GPa, *C*_1_=-0.000895 GPa, *b*_1_=-2, *C*_2_=0.002101 GPa, *b*_2_=-1, *C*_3_=0.000115 GPa, *b*_3_=-4 |
| Annulus fibrosus fiber lamina | | Shell | Fabric | Load curve used, material angle *β*=±65, ±60, ±50, ±45 for 8 layers |
| Upper thoracic ligaments (T1-T6) | Anterior Long. | Beam | Elastic spring discrete beam | TDF=8.1 mm, FF=0.173 kN |
|  | Posterior Long. |  |  | TDF=4.2 mm, FF=0.093 kN |
|  | Ligamenta-Flava |  |  | TDF=7.4 mm, FF=0.155 kN |
|  | Interspinous |  |  | TDF=8.5 mm, FF=0.063 kN |
|  | Supraspinous |  |  | TDF=12.3 mm, FF=0.189 kN |
|  | Intertrans (left & right) |  |  | TDF=5.8 mm |
|  | Facet capsular (left & right) |  |  | TDF=6.1 mm, FF=0.214 kN |
| Lower thoracic ligaments (T7-T12) | Anterior Long. | Beam | Elastic spring discrete beam | TDF=12.7 mm, FF=0.353 kN |
|  | Posterior Long. |  |  | TDF=5.1 mm, FF=0.098 kN |
|  | Ligamenta-Flava |  |  | TDF=9.1 mm, FF=0.227 kN |
|  | Interspinous |  |  | TDF=5.3 mm, FF=0.063 kN |
|  | Supraspinous |  |  | TDF=15 mm, FF=0.221 kN |
|  | Intertrans (left & right) |  |  | TDF=6.5 mm |
|  | Facet capsular (left & right) |  |  | TDF=6.1 mm, FF=0.214 kN |
| Costovertebral joint ligaments | Radiate | Shell | Fabric | *E*_a_=0.046 GPa (T2-T5), 0.024 GPa (T6-T9), 0.049 GPa (T1, T10-T12) |
|  | Superior costotransverse | Beam | Elastic spring discrete beam | FF=0.0491 kN |
|  | Costotransverse | Beam | Elastic spring discrete beam | FF=0.0365 kN (T2-T5), 0.0879 kN (T6-T9), 0.0896 kN (T10) |
|  | Lateral costotransverse | Beam | Elastic spring discrete beam | FF=0.0204 kN (T2-T5), 0.0363 kN (T6-T9), 0.0655 kN (T10) |
|  | Intra-articular | Beam | Elastic spring discrete beam | FF=0.209 kN (T2-T5), 0.189 kN (T6-T9) |

Notes: *E*: Young’s Modulus, *σ*_Y_: Yield Stress, *E*_t_: Tangent modulus, *E*_a_: Young’s modulus (longitudinal direction), *K*: Bulk modulus, *C*_i_ and *b*_i_: Material constants for Hill foam material, TDF: Tensile displacement at failure, and FF: Maximum tensile force before failure.

**SM-B: Overview of the steps of the study.**

| **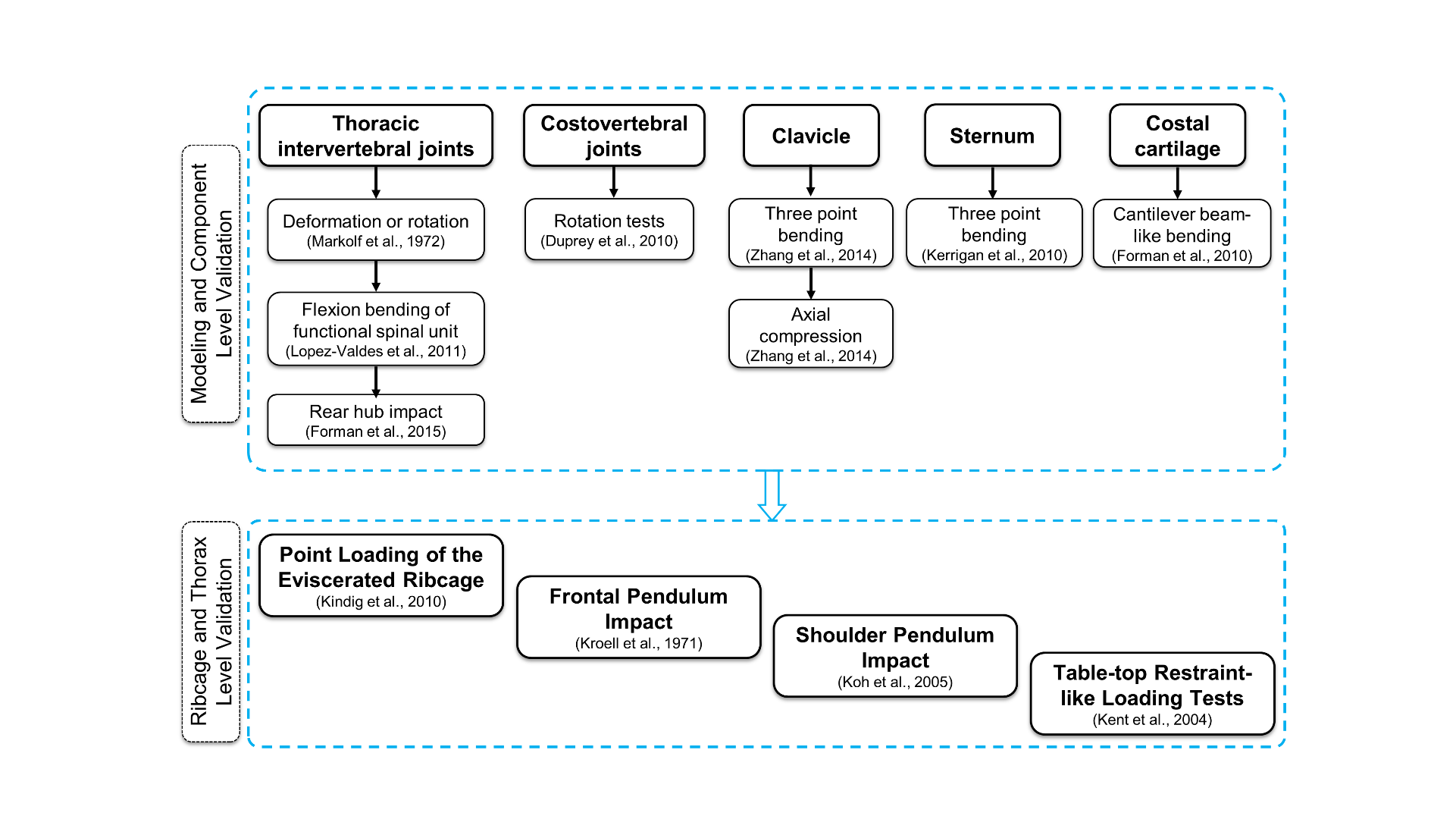** |
| --- |
| Figure 1. Overview of the modeling and validation tests implemented in current study. |

**SM-C: Model setup for the costovertebral joints.**

| 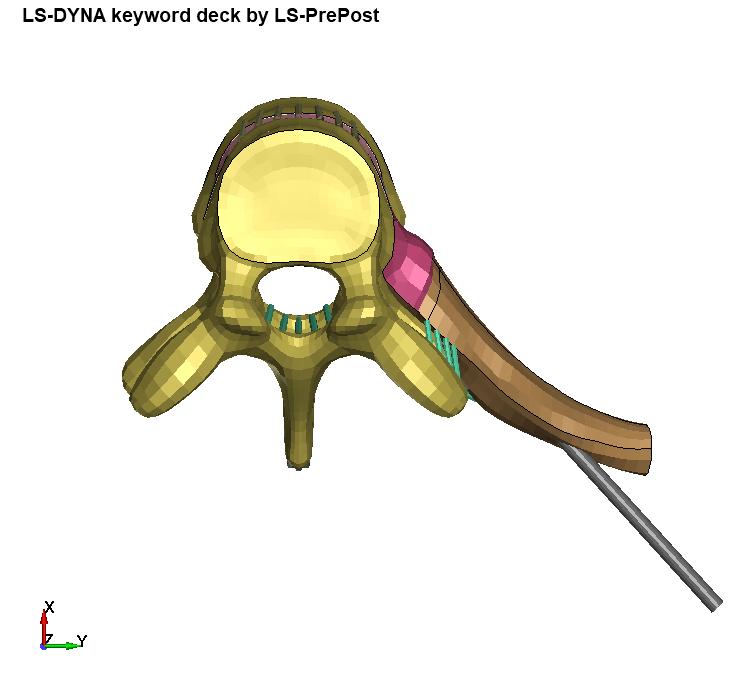 Y  Z  *Cranial-caudal flexion*  *Ventral-dorsal flexion*  *Fixed*  *Rod* |
| --- |
| Figure 2. Model setup for quasi-static rotation of the costovertebral joints. |

**SM-D: Deformation of the thoracic intervertebral joints in response to external loads.**

| 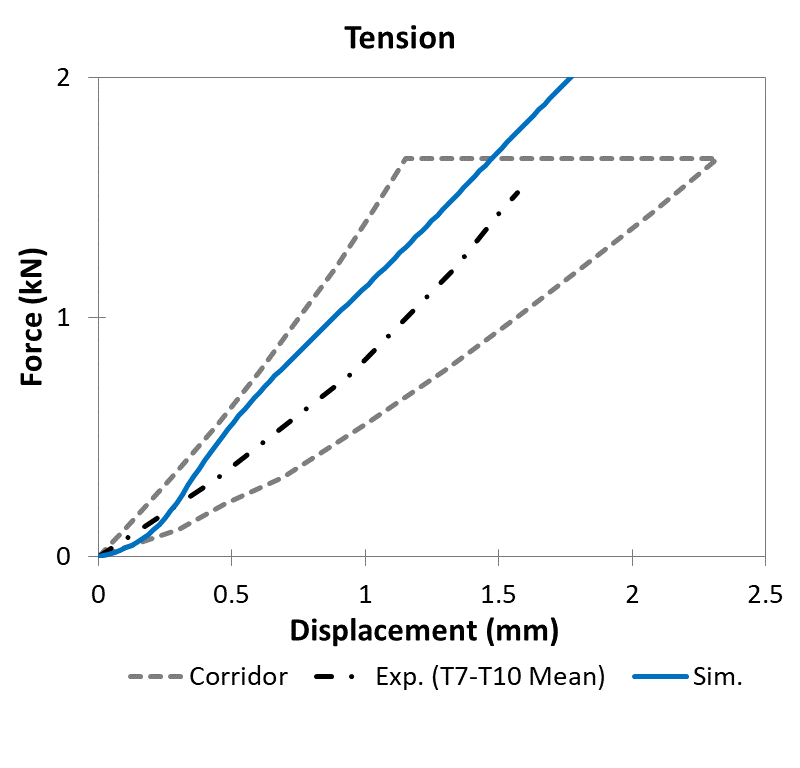 | 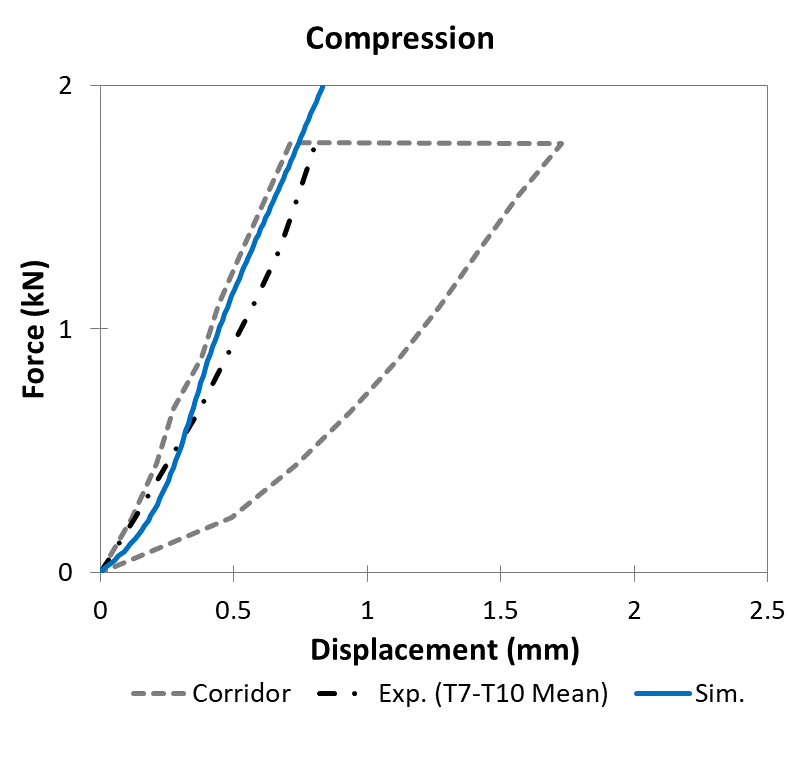 |
| --- | --- |
| (a) | (b) |
| 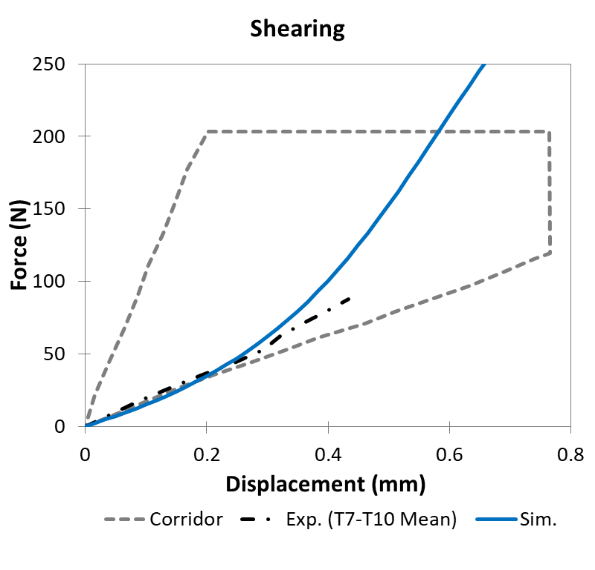 | 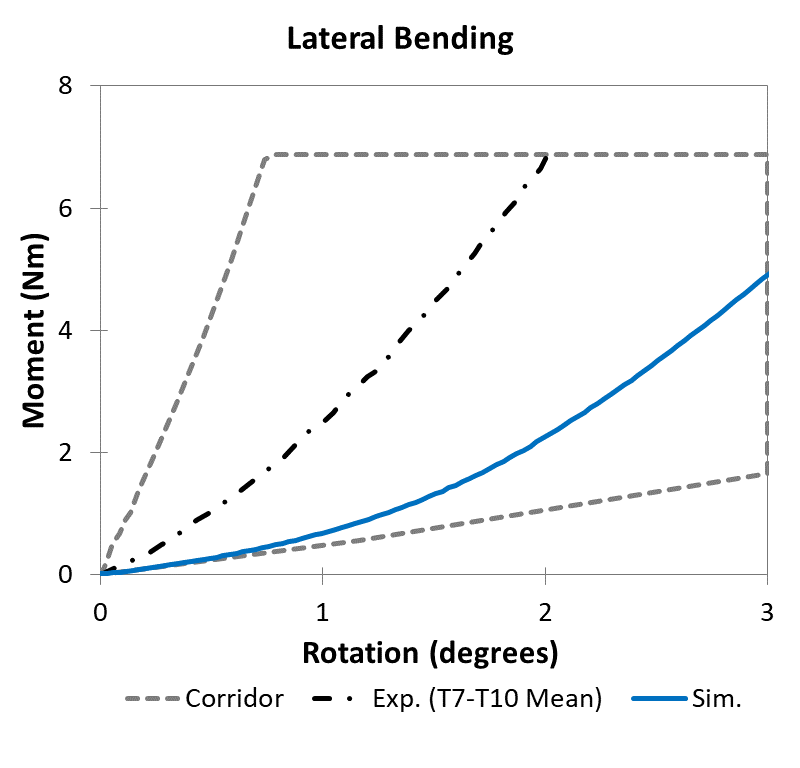 |
| (c) | (d) |
| Figure 3. FE simulation results for adjacent vertebrae T8-T9: force vs. displacement responses under (a) tension, (b) compression, and (c) shearing, and (d) moment vs. rotation response under lateral bending. | |

**SM-E: Costovertebral joints evaluation results.**

| 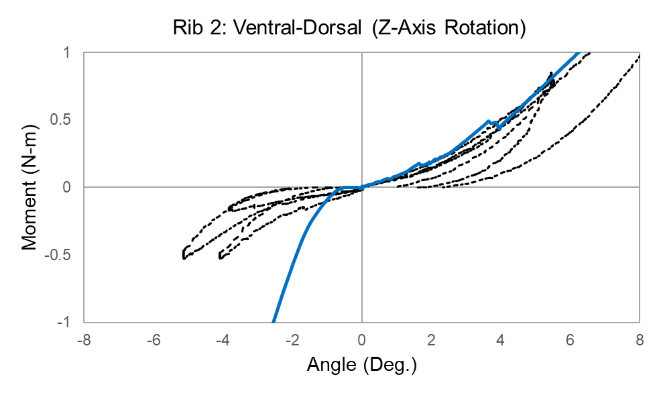 | 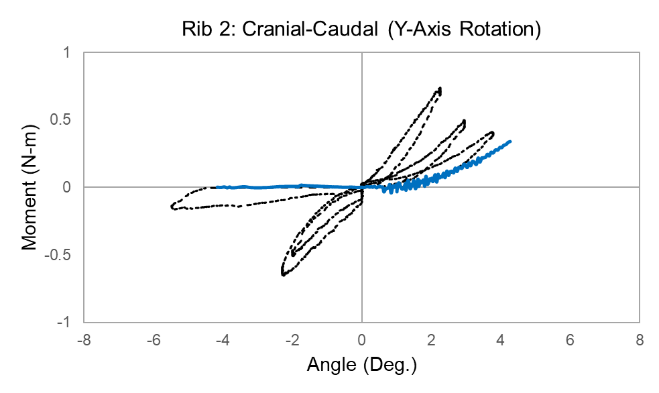 |
| --- | --- |
| 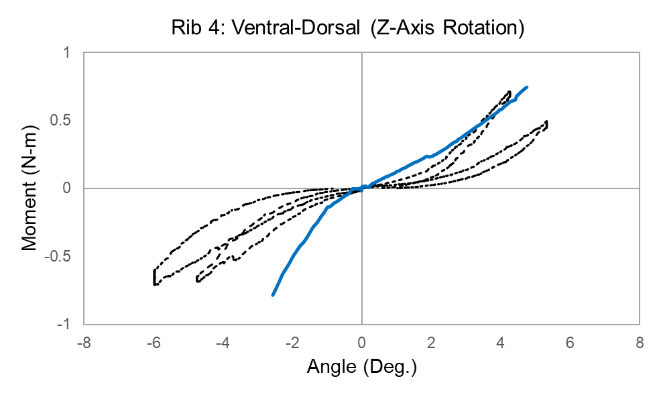 | 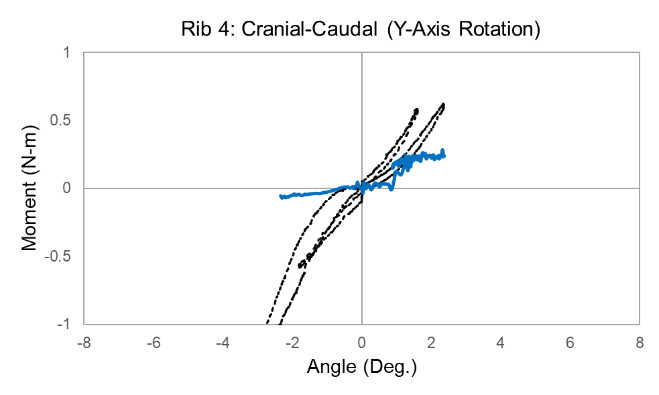 |
| 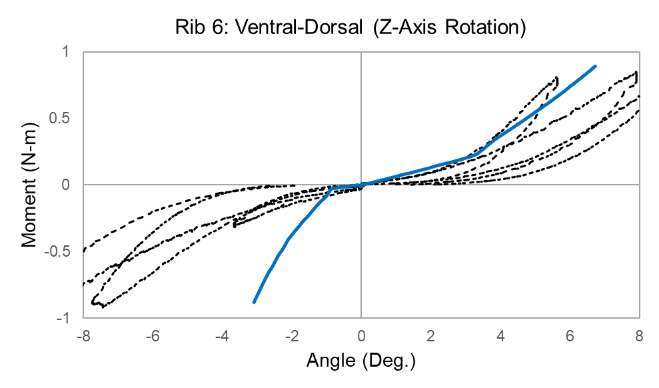 | 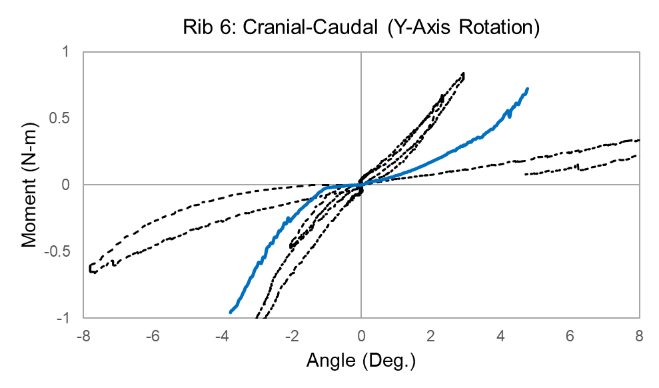 |
| 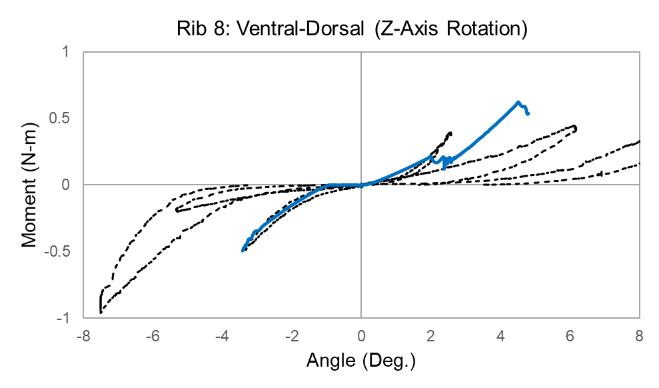 | 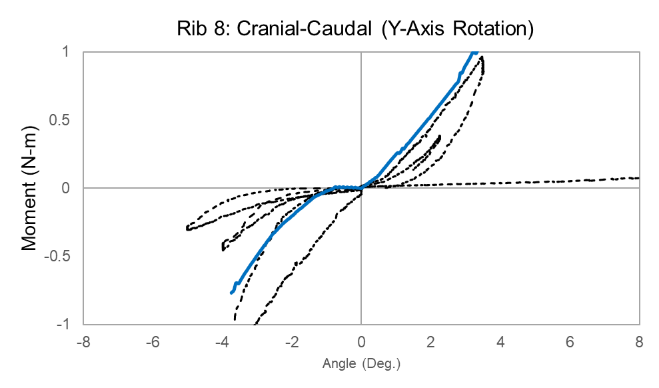 |
| 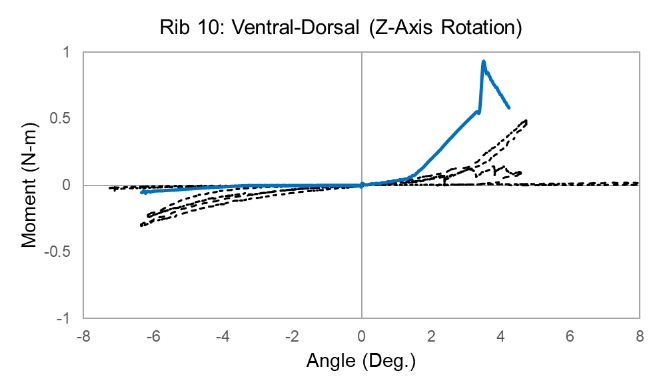 | 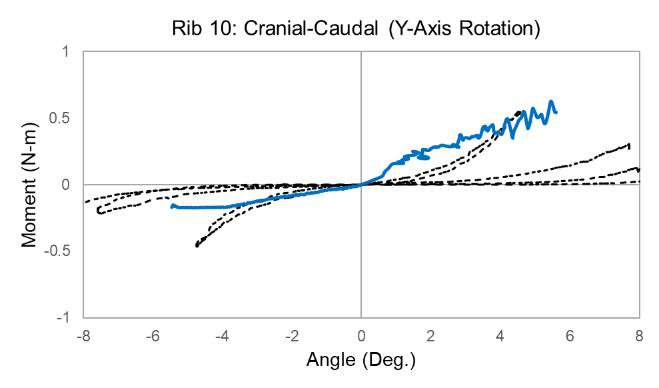 |
| Figure 4. FE simulation results for costovertebral joints evaluation at multiple levels (ribs 2, 4, 6, 8, and 10): ventral-dorsal flexion and cranial-caudal flexion. | |

**SM-F: Clavicle model validation results.**

| 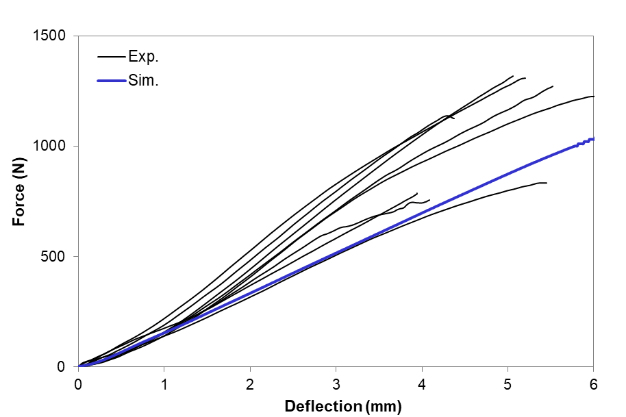 | 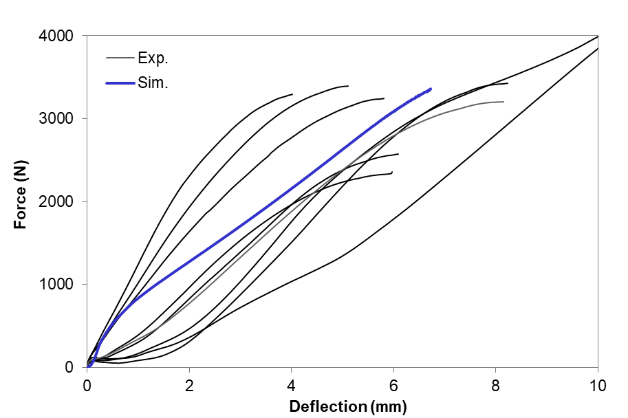 |
| --- | --- |
| (a) | (b) |
| 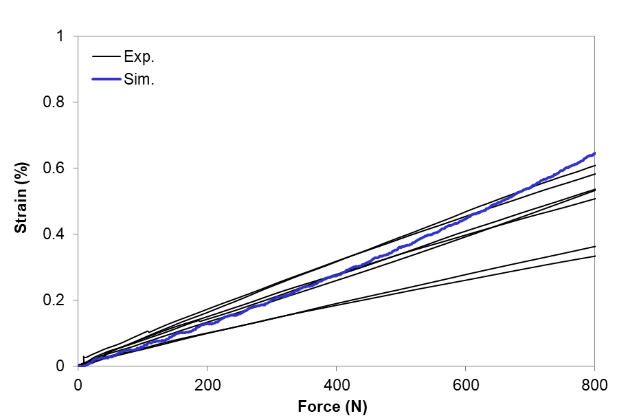 | 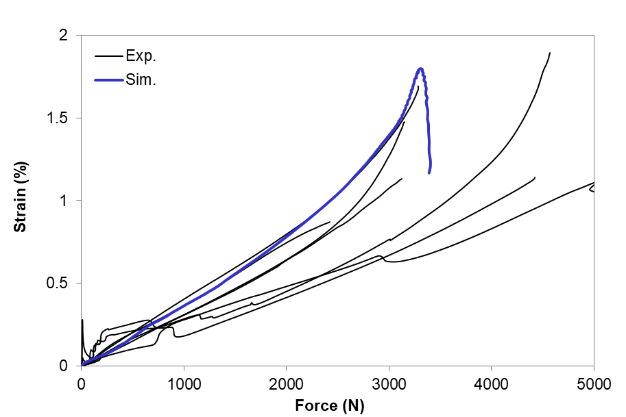 |
| (c) | (d) |
| Figure 5. FE simulation results for clavicle model evaluation: (a) force vs. deflection response under three-point bending, (b) force vs. deflection response under axial compression, (c) strain vs. force response under three-point bending, and (d) strain vs. force response under axial compression. | |

**SM-G: Sternum model validation results.**

| 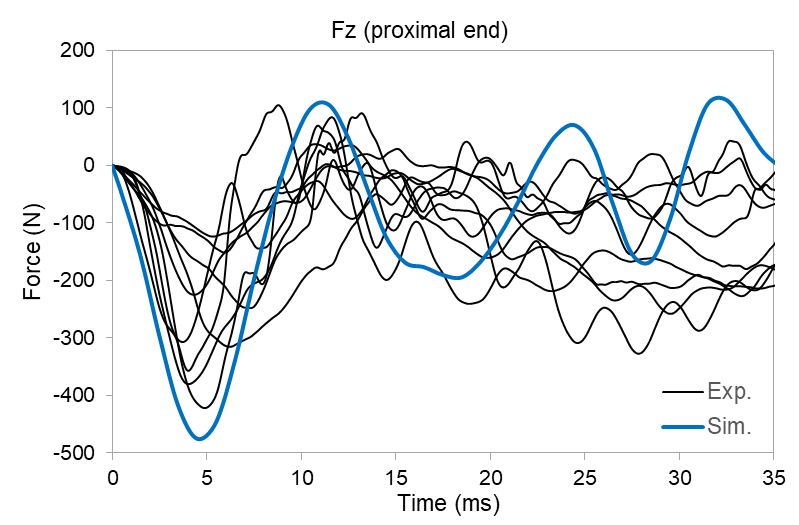 | **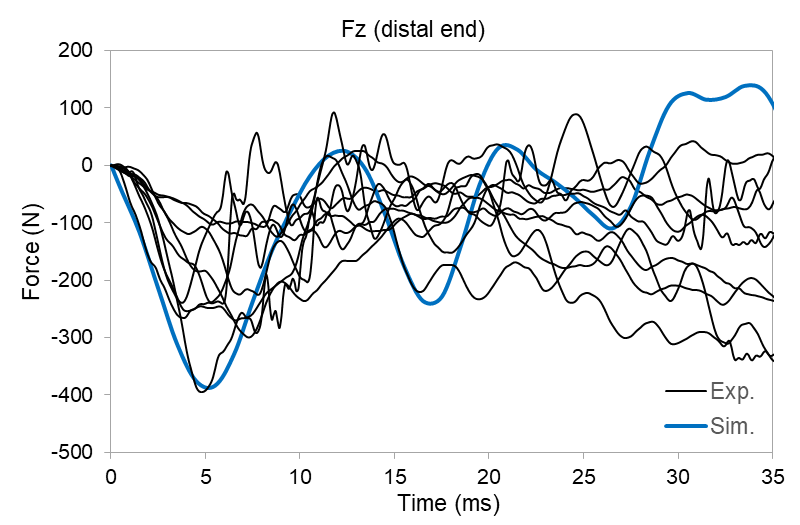** |
| --- | --- |
| (a) | (b) |
| 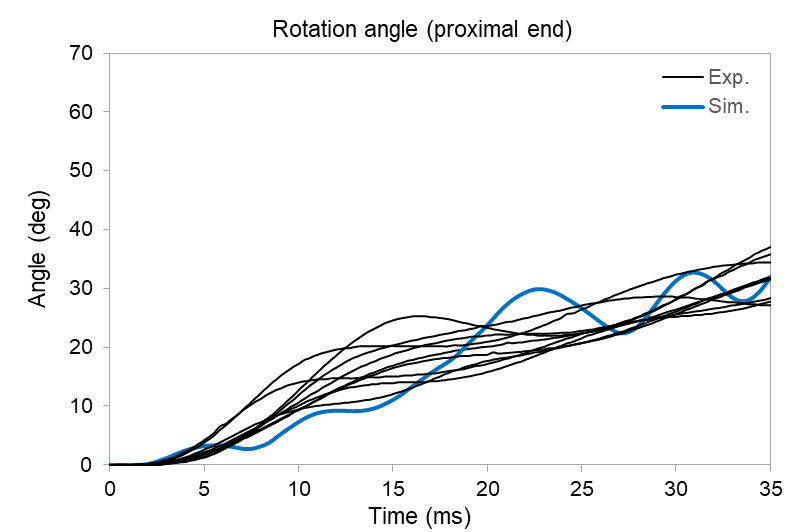 | 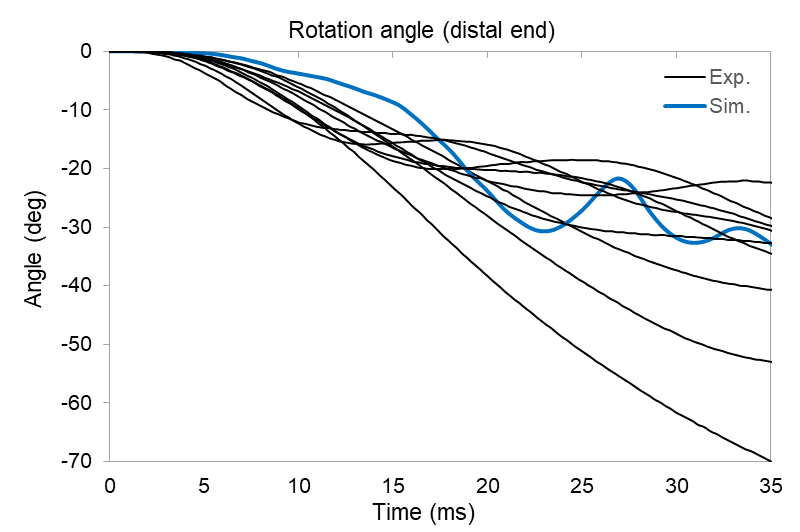 |
| (c) | (d) |
| Figure 6. FE simulation results for sternum model evaluation under three-point bending testing: (a) proximal end force (in vertical direction based on testing device) vs. time, and (b) distal end force (in vertical direction based on testing device) vs. time. | |

**SM-H: Costal-cartilage model validation results.**

| 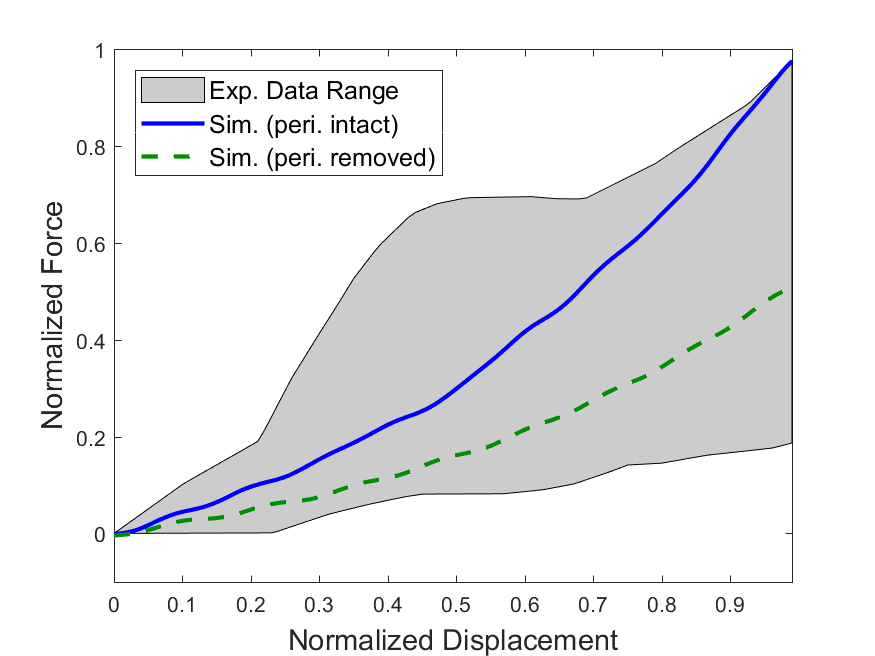 |
| --- |
| (a) |
| 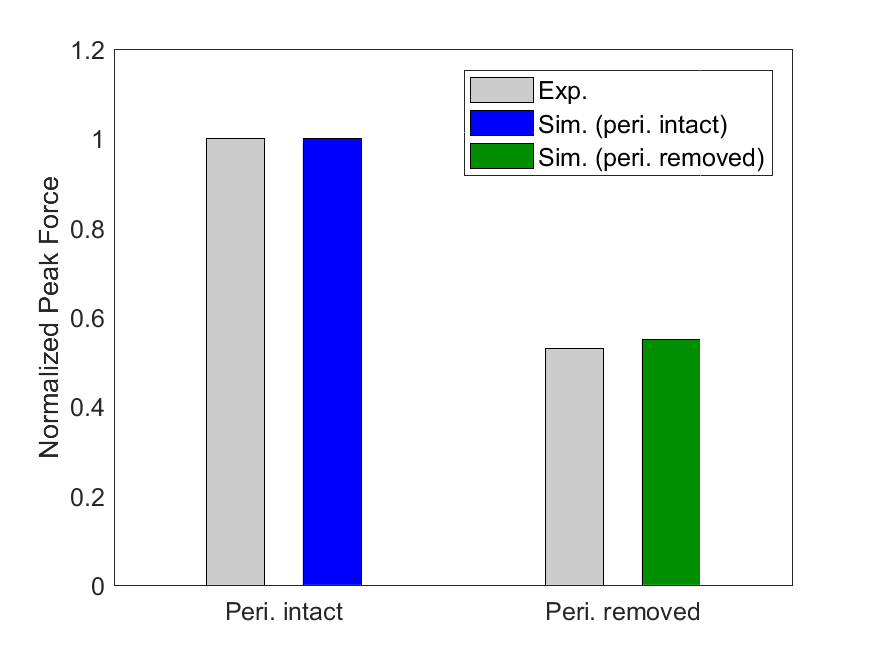 |
| (b) |
| Figure 7. Costal-cartilage FE simulation results compared with experiments: (a) a comparison of the force-displacement response of the FE model against the corridor data (Forman et al., 2010), and (b) a bar graph comparing the average normalized peak forces for both perichondrium intact and removed cases. |
